# Potential of Saliva in Stroke Patients: A Review

**DOI:** 10.3390/ijms27156871

**Published:** 2026-07-31

**Authors:** Dominika Forszt, Karolina Gerreth, Marzena Bielas, Mateusz Maciejczyk

**Affiliations:** 1 Department of Risk Group Dentistry, Chair of Pediatric Dentistry, Poznan University of Medical Sciences, 60-812 Poznan, Greater Poland Voivodeship, Poland; dforszt@ump.edu.pl (D.F.); karolinagerreth@ump.edu.pl (K.G.); 2 Department of Family Medicine, Poznan University of Medical Sciences, 60-512 Poznan, Greater Poland Voivodeship, Poland; mbielas@ump.edu.pl; 3 Department of Hygiene, Epidemiology and Environmental Health, Medical University of Bialystok, 15-222 Bialystok, Podlaskie Voivodeship, Poland

**Keywords:** stroke, saliva, markers, salivary biomarkers, inflammation, oxidative stress

## Abstract

Cerebral strokes have been a global problem for many decades. Interest in saliva as a diagnostic material has grown substantially. The ease of collection, minimal invasiveness and low storage costs make saliva particularly well suited for diagnostic and therapeutic applications. Research shows that markers detected in blood can also be found in saliva, opening up the possibility of using this biofluid for clinical purposes. The aim of this paper is to review current knowledge on salivary biomarkers in stroke patients. Among the markers detected in the saliva of stroke patients are indicators of antioxidant capacity (e.g., reduced glutathione and uric acid), oxidative (e.g., malondialdehyde, advanced oxidation protein products) and nitrosative stress (e.g., peroxynitrite), inflammation (pro- and anti-inflammatory cytokines) and others. The levels of many salivary biomarkers correlate with the severity of disease, organ complications and may serve as valuable tools for the differential stroke diagnosis. The collective findings of these studies underscore the potential clinical applications of salivary biomarkers in stroke. However, considerable obstacles such as methodological heterogeneity, limited differential diagnostic studies, small cohorts, and insufficient standardization of saliva collection and oral health assessment impede their clinical implementation. Establishing standardized saliva collection protocols and reference values for key parameters is crucial. Identifying biomarkers in saliva lays the groundwork for developing innovative tools to analyze and accurately assess stroke patients.

## 1. Introduction

Diagnostic biomarkers can be detected in a variety of body fluids, including saliva, blood, urine, and cerebrospinal fluid. Saliva, in particular, is gaining popularity as a diagnostic medium due to its non-invasive collection, ease of handling and simple storage requirements, making it an increasingly valuable tool in clinical testing [1]. The presence of many biomarkers in both blood and saliva underscores saliva’s potential as a reservoir of molecules that reflect the patient’s current health status [2]. Changes in salivary composition have been observed in patients with conditions such as diabetes [3], chronic kidney disease [4], cancer [5], atherosclerosis [6], myocardial infarction [7], chronic heart failure [8], and other systemic diseases.

Stroke is a civilization disease, characterized by the obstruction of blood flow to specific regions of the brain, resulting in reduced oxygen and nutrient supply and subsequent pathological changes in the brain. There are two main types of stroke: ischemic stroke, caused by occlusion of cerebral blood flow, and hemorrhagic stroke, resulting from vessel rupture and subsequent extravasation of blood into the brain [9]. Neuronal ischemia induces a number of processes including necrosis, neuroinflammation, excitotoxicity, apoptosis, and free radical formation [10]. Following stroke, disruption of the blood-brain barrier (BBB) permits the passage of molecules into the systemic circulation, where they can be detected in body fluids such as blood and saliva [11]. Among the markers found in the saliva of stroke patients are those reflecting antioxidant capacity, oxidative stress, nitrosative stress and inflammation [12,13]. Therefore, saliva can be a valuable resource in the diagnosis and monitoring of stroke. This paper aims to review the current knowledge on the diagnostic significance of salivary biomarkers in stroke patients.

Stroke is caused by compromised blood flow to the brain. An ischemic stroke happens when the blood supply to the brain is blocked (i.e., cerebrovascular obstruction by thrombosis or emboli). A hemorrhagic stroke occurs when a blood vessel in the brain ruptures (due to hypertension, aneurysm, or arteriovenous malformation). This results in a decreased supply of oxygen and nutrients to the brain, and consequently in neuronal injury and death [14,15]. Of all strokes, 87% are ischemic, and 13% are hemorrhagic (10% ICH, 3% SAH) [16]. The primary risk factor for stroke is high systolic blood pressure (SBP; accounting for 56.8% of cases), followed by ambient particulate matter pollution (16.6%), smoking (13.8%), high body mass index (BMI) (24%), high LDL cholesterol (13.1%), household air pollution (11.2%), diet high in sodium (10.6%) and high fasting plasma glucose (FPG; 10.3%) [17]. Other factors that can play a role in stroke genesis include: age over 35 years, male sex, ethnicities such as Black, African, American and Hispanic, diabetes, dyslipidemia, inflammatory states, atrial fibrillation, hypercoagulability, hormone replacement therapy, alcohol use and lack of physical activity [18]. In younger patients, causes include cryptogenic (24–53%), cardio-embolism (10–34%), large artery atherosclerosis (4–29%), or small vessel disease (12–26%). Among young patients with cryptogenic stroke, 40–56% have patent foramen ovale (PFO). Many of these individuals also have hyperlipidemia, migraine with aura or a history of hormonal contraception use—factors that may interact with PFO to increase stroke risk [18].

Stroke is diagnosed through a comprehensive neurological examination, detailed medical history, and neuroimaging studies [19]. Quick diagnostic tests are currently unavailable. Their absence underscores the need to develop rapid tests that optimize patient management and improve stroke outcomes. As described by the Biomarkers Definition Working Group, a biomarker is a measurable molecule used to assess normal biological activities, disease progression, or the effects of therapeutic interventions [20]. Each biomarker should meet key criteria, including high sensitivity and specificity, noninvasiveness, cost-effectiveness and ease of sample collection [21]. The plausibility and reproducibility of results are essential considerations [22]. Stroke biomarkers should differentiate true strokes from mimics, clarify stroke etiology, and predict severity and outcomes, including early neurological deterioration and hemorrhagic complications, while identifying patients who may benefit from targeted interventions such as decompressive hemicraniectomy or arterial recanalization [23]. The search for stroke-specific biomarkers focuses on brain-derived molecules released following BBB damage [24]. Redox and inflammatory biomarkers are of primary interest because oxidative stress and increased cytokine production play a central role in the pathogenesis of stroke.

Most of the saliva is produced by three pairs of glands, i.e., sublingual, submandibular, and parotid glands. The other sources of this biofluid are oro-naso-pharyngeal secretions, minor salivary glands (localized on the palate, buccal mucosa, and tongue), as well as the gingival crevicular sulci [25]. The salivary glands consist of acini, in which the primary or initial saliva is formed [25]. Parotid glands secrete serous saliva, which is rich in amylase, sialomucins and sulfomucins [26]. Submandibular and sublingual glands are composed of acini that produce both serous and mucous saliva [27]. There is a predominance of serous-producing acinar cells in the submandibular glands, and mucous producing cells in the sublingual glands [28]. There are 600–1000 minor salivary glands in the oral cavity [29]. Their sizes range from 1–5 mm [30]. They are distributed throughout the submucosa of the oral cavity, except in the gingiva and hard palate [26]. Minor salivary glands produce immunoglobulins, lysozymes, and salivary acid phosphatase, and are responsible for approximately 70% of salivary mucin production. They play a critical role in protecting tooth surfaces and oral mucosa [26]. The innervation of the salivary glands comes from both the sympathetic and parasympathetic nervous systems [31]. Sympathetic stimulation triggers the secretion of protein-rich saliva [31], via the superior cervical ganglion, whose fibers extend to all salivary glands through the external carotid plexus and its branches [32]. Parasympathetic stimulation produces watery, ion-rich saliva [28]. In the case of the parotid gland, preganglionic parasympathetic fibers originate in the inferior salivatory nucleus of the medulla and reach the otic ganglion via the glossopharyngeal nerve [32].

Up to 2 L of saliva can be produced daily [33]. Unstimulated salivary flow ranges from 0.3–0.4 mL/min, while stimulated flow ranges from 1.5–2.0 mL/min [34]. The submandibular gland contributes primarily to the production of unstimulated saliva, accounting for approximately 65%, while the parotid gland produces over 50% of stimulated saliva [35].

Salivary molecules originate either from the bloodstream or are synthesized directly by the salivary glands [36]. BBB is the border between brain tissue and blood [37,38]. The barrier consists of a basement membrane, endothelial cells, pericytes and astrocytes [39]. The components of the BBB together with the neurons and the extracellular matrix (ECM) form the neurovascular unit [40,41]. The basal membrane contains type IV collagen, laminin, fibronectin or heparan sulfate [42]. The endothelial cells that make up the vessel wall are connected by adherens junctions (AJs) and tight junctions (TJs) [40,43]. Damage to BBB occurs in two phases [40,44]. Increased permeability is primarily mediated by matrix metalloproteinases (MMPs) [45], which degrade collagen within the basement membrane and disrupt proteins constituting tight junctions [42]. Oxidative stress indirectly enhances MMP activity [45]. Peroxynitrite (ONOO^−^) induces neurotoxicity through the activation of MMP-1 (matrix metalloproteinase 1), MMP-2 (matrix metalloproteinase 2), and MMP-9 (matrix metalloproteinase 9) [41]. MMPs are secreted by microglia, endothelial cells, and astrocytes [46]. According to Lakhan et al. [47]., MMPs may also be secreted by neurons. Pericytes maintain TJ integrity by releasing pro-angiogenic factors that promote the synthesis of TJ–building proteins [45]. Free radicals and inflammation trigger pericyte contraction [48] and endothelial damage, disrupting BBB’s compact structure and increasing its permeability [49]. Chemokine-driven leukocyte influx is associated with the release of proteases, pro-inflammatory factors, reactive oxygen species (ROS) and MMPs, which in turn cause secondary BBB damage [42]. Increased BBB permeability allows molecules to pass from the brain into the circulation [50]. Clinically, these molecules can be detected in bodily fluids as markers of stroke [51].

The passage of biomarkers from blood to saliva occurs via both intracellular and extracellular transport mechanisms [52]. Small molecules can penetrate passively via diffusion, navigating five sequential barriers: the blood vessels walls, the interstitial space and subsequently the basal membrane, cytoplasm and luminal membrane of acinar or duct cells [53]. Cortisol, for example, enters saliva via passive transport [54,55]. In contrast, active transport, a form of transcellular movement, facilitates the secretion of Immunoglobulin A (sIgA) [56]. Additionally, molecules can enter saliva through gaps between the acinar and ductal cells via a process called ultrafiltration [57]. However, biomarker concentrations in saliva can be 300 to 3000 times lower than in blood [56]. Molecules enter saliva not only through the secretory activity of the salivary glands but also via gingival crevicular fluid and the oral, nasal, and pharyngeal mucosa [57]. Key pro-inflammatory factors include interleukin-6 (IL-6), tumor necrosis factor alpha (TNF-ɑ) and c-reactive protein (CRP) [58]. CRP is a protein whose blood levels rise in response to both local and systemic inflammation. It has been hypothesized that CRP enters saliva via gingival crevicular fluid (GCF), as its molecular size prevents passage through simple diffusion or ultrafiltration [59]. In contrast, TNF-ɑ and IL-6 may enter saliva from the bloodstream via ultrafiltration and can also be produced locally by the salivary glands and lymphoid cells [60].

Saliva is attracting growing interest in laboratory medicine [2,61,62,63] as a promising diagnostic material and a potential alternative to blood and cerebrospinal fluid. Saliva samples are noninvasive, highly stable, and easy to collect, transport, and analyze, making this biofluid particularly useful for clinical applications [2,61,63]. Salivary biomarkers may play a pivotal role in future. Previous reviews have highlighted the potential of salivary biomarkers in neurological and neuropsychiatric disorders [11,63,64,65]. However, to the best of our knowledge, no review has comprehensively summarized the current evidence on salivary biomarkers in stroke. The present review is focused specifically on stroke and provides a comprehensive overview of salivary biomarkers across this specific disease entity. It encompasses a broad spectrum of biomarkers and summarizes evidence accumulated over more than two decades of research. By integrating findings from studies published over the last 20 years, this review offers detailed and extensive knowledge on this topic that distinguishes it from reviews addressing various neurological or neuropsychiatric conditions or focusing on selected biomarker groups.

## 2. Redox Biomarkers

Under physiological conditions, brain hypoxanthine is sequentially converted to xanthine and then to uric acid (UA) through the action of xanthine dehydrogenase [66]. During stroke, ischemia and hypoxia trigger proteolytic conversion of xanthine dehydrogenase into xanthine oxidase (XO) [67,68]. In addition to hypoxanthine, XO utilizes oxygen (O_2_) as a substrate, generating superoxide anions in the process [67]. Thus, XO activation leads to ROS overproduction, as the enzyme generates high amounts of superoxide radicals and H_2_O_2_ [68]. Upon BBB disruption, XO may enter the circulation and reach the saliva via ultrafiltration, through compromised cellular membranes, or by local synthesis in the salivary glands [69].

Research on biomarkers of oxidative stress in saliva in stroke patients are few and limited in scope. In our previous study we evaluated salivary XO and its enzymatic products, UA and hydrogen peroxide (H_2_O_2_), in patients with ischemic and hemorrhagic stroke and healthy controls. Compared with controls, both stroke subgroups showed increased XO activity and UA levels, whereas H_2_O_2_ concentrations were elevated mainly in ischemic stroke patients [69]. Furthermore, XO-specific activity was inversely correlated with cognitive and functional outcomes, as measured by postural balance (Berg Balance Scale [BBS]), performance in activities of daily living (Barthel Index [BI] for Activities of Daily Living [ADL]) and global cognition (Addenbrooke’s Cognitive Examination III [ACE-III]) [69]. BI is a widely used tool for assessing performance in ADL and overall functional independence. Higher BI scores correspond to greater independence in daily activities [70]. BBS assesses a patient’s ability to maintain balance during specific tasks offering an objective measure of walking and postural recovery, particularly in stroke patients [71]. ACE-III is a sensitive tool for the early detection of cognitive impairment, evaluating five key cognitive domains to provide a comprehensive assessment of cognitive status [72]. Notably, XO-specific activity was negatively correlated with all cognitive domains, including memory, orientation, language, attention, visual perception, and visuospatial functions. XO-specific activity was shown to distinguish stroke survivors with mild cognitive impairment (ACE III scores 61–88) from those with moderate cognitive impairment (ACE III scores < 61) with a 100% sensitivity and 93.75% specificity. However, it could not distinguish patients with no cognitive decline from those with mild cognitive impairment [69].

Nitric oxide (NO) is generated through the oxidation reaction of arginine [73] and plays a key role in initiating salivary secretion [74]. Under conditions of oxidative stress, NO depletion occurs as it reacts with oxygen free radicals, forming the highly reactive peroxynitrite (ONOO^−^) [39,75,76]. Maciejczyk et al. [61] investigated NO and ONOO^−^ levels in stroke patients with normal salivary secretion or hyposalivation. Stroke patients with hyposalivation exhibited significantly lower NO levels in NWS than those with normal salivation and healthy controls. In contrast, ONOO^−^ concentrations were increased in both stroke subgroups, with the highest levels observed in patients with hyposalivation. These findings suggest that enhanced oxidative stress promotes the conversion of NO to ONOO^−^, contributing to impaired salivary gland function and hyposalivation [61]. To date, no further studies on this topic have been published, and a thorough understanding of the role of nitrosative stress in salivary gland dysfunction following a stroke is essential.

Glycation is a non-enzymatic, post-translational modification of proteins that results in the formation of advanced glycation end-products (AGEs) [77]. AGEs arise via the Maillard reaction, in which reducing sugars react with amines, forming an Amadori product as a key intermediate [78]. Protein glycation is closely intertwined with protein oxidation, a process that encompasses multiple oxidative modifications. These include formation of carbonyl derivatives, oxidation of sulfur containing amino acids, chlorination of amino groups, hydroxylation of aliphatic and aromatic groups, tyrosine nitration, and cysteine nitrosylation or glutathionylation [79]. Protein carbonyls formed when free radicals oxidize protein backbones and amino acid residues such as proline, arginine, lysine, and threonine [80], serve as widely used biomarkers for assessing protein oxidative damage [81]. Under conditions of redox imbalance, tryptophan can be converted into kynurenine and N-formylkynurenine [82]. Additionally, advanced oxidation protein products (AOPPs) are clusters of abnormal proteins that typically show extensive halogenation of dityrosine or lysine residues [83].

Research on salivary biomarkers of protein glycation in stroke patients is also limited. In our previous study we demonstrated significantly higher levels of AGEs and Amadori products in stroke patients with hyposalivation than in those with normal salivation and healthy controls [61]. Patients with hyposalivation also exhibited increased oxidative and carbonyl stress, reflected by elevated salivary protein carbonyls, nitrotyrosine, dityrosine, N-formylkynurenine, and kynurenine, mainly in NWS. Additionally, RNS appeared to affect salivary gland function, as ONOO^−^ and nitrotyrosine levels were negatively correlated with secretory function. Total thiols, tryptophan, and total protein concentrations were significantly reduced in the hyposalivation subgroup, indicating impaired antioxidant defense and salivary gland dysfunction [61]. Accumulation of these protein modification products may impair salivary gland secretory function and alter saliva composition. Such deposits could affect secretory cells or the vasculature of salivary glands, potentially increasing tissue stiffness [61]. In a subsequent study, the same research group reported significantly decreased total protein concentrations in both NWS and stimulated-whole saliva (SWS) among patients divided into ischemic and hemorrhagic stroke subgroups, compared with controls [69]. Nonetheless, additional research is necessary to elucidate the mechanisms by which oxidative and nitrosative stress and protein glycation drive salivary gland hypofunction in these patients. An important strength of both studies was the detailed methodology, including comprehensive inclusion and exclusion criteria, standardized saliva collection, as well as dental and periodontal examination with well-matched healthy controls. However, the studies were limited by their relatively small sample size, single-center design, inclusion of only patients in the subacute phase of stroke, and cross-sectional assessment based on a single saliva collection. It is worth noting that the division into patients with hyposalivation and normosaliviation indicates the direction of research aimed at salivary gland dysfunction rather than differential diagnosis.

Gerreth et al. [13] analyzed salivary redox status, reflecting the dynamic interplay between antioxidants and free radicals [84]. Stroke induces excessive free radical production that overwhelms antioxidant defenses, resulting in oxidative stress [85]. Assessing redox status is challenging due to the complexity of its components [86]. Consequently, measurements of total antioxidant capacity (TAC), total oxidant status (TOS) and oxidative stress index (OSI) are increasingly employed. TAC, in particular, provides an integrated assessment of both enzymatic and non-enzymatic antioxidants [86]. TAC includes antioxidants that are difficult to measure, as well as ones that remain unidentified [87]. TOS reflects the overall oxidant properties of the sample [82]. OSI, calculated as the ratio of TOS to TAC, indicates whether the redox balance is shifting toward oxidation [88]. Gerreth et al. [13] compared these salivary redox biomarkers in stroke survivors and age- and sex-matched healthy controls [13]. Although TAC did not differ significantly between the groups, both salivary TOS and OSI were elevated, indicating increased oxidative stress in stroke patients [13]. The rise in TOS reflects enhanced oxidative processes, and the elevated OSI indicates a shift in redox balance toward oxidation. No further studies on these topics exist.

Enzymatic antioxidants serve as the first line of defense against oxidative stress. In saliva, the primary enzymatic antioxidants are superoxide dismutase (SOD), catalase (CAT), and peroxidase (Px), which play central roles in maintaining redox balance [64]. SOD catalyzes the conversion of superoxide radicals to hydrogen peroxide (H_2_O_2_) [89,90], which is subsequently broken down by catalase into water and molecular oxygen [91,92], using iron (Fe) as a cofactor to prevent excessive H_2_O_2_ accumulation [90]. Although salivary peroxidase constitutes only about 0.01% of the total salivary protein content, it is a critical component of the salivary antioxidant system, exerting a disproportionately significant protective effect [92,93]. Peroxidases (Px) are a class of oxidoreductases that reduce peroxides, including H_2_O_2_, while simultaneously oxidizing organic and inorganic substrates [94]. In the presence of thiocyanate ions (SCN^−^) and H_2_O_2_, salivary Px catalyzes the formation of hypothiocyanous acid and the hypothiocyanite anion through the oxidation of SCN^−^. These products exhibit antibacterial activity and contribute to the removal of excess H_2_O_2_, thereby supporting both antimicrobial defense and redox balance [94].

Investigation of salivary SOD, CAT and Px by Gerreth et al. [13] revealed a marked increase in stroke patients, suggesting an adaptive response of the salivary glands to elevated ROS by enhancing enzymatic antioxidant defenses. Al-Rawi et al. [95,96] also conducted two studies examining enzymatic antioxidant defenses in the serum and NWS of stroke survivors. Each study included 50 patients with clinically and radiologically confirmed ischemic stroke and 25 healthy controls [95,96]. In another study, participants were further divided into three groups according to the presence of comorbidities associated with increased stroke risk, including hypertension, type 2 diabetes, and ischemic heart disease [95]. In this study, salivary and serum SOD concentrations were significantly higher in stroke patients than in healthy controls. Notably, salivary SOD levels were also elevated in hypertensive and ischemic heart disease patients within the stroke-risk group [95].

Iron (Fe), a cofactor for antioxidant enzymes [97], is also released from hemoglobin breakdown during brain injury [98]. García-Cabo et al. [99] measured salivary Fe in 20 patients, evenly divided into ischemic and hemorrhagic stroke subgroups. Nasal exudate and non-stimulated whole saliva (NWS) were analyzed. Fe levels were significantly higher in nasal exudate from hemorrhagic than ischemic stroke but did not differ in NWS [99]. Inclusion of healthy controls could provide additional insights.

When enzymatic defenses are depleted, low-molecular-weight antioxidants assume the primary protective role. Endogenous non-enzymatic antioxidants include albumin, bilirubin, glutathione, uric acid, ceruloplasmin, transferrin, and coenzyme Q10 [100]. Exogenous antioxidants include vitamins A, C, and E, carotenoids, polyphenols and xanthophylls [101]. These compounds act by neutralizing reactive oxygen species (ROS) [88,101,102,103,104], and suppressing lipid peroxidation, thereby limiting the generation of additional free radicals [91,103,105,106,107].

Uric acid (UA) exhibits strong antioxidant properties, accounting for approximately 70% of total antioxidant activity in saliva and exerts neuroprotective effects in the brain [108,109,110]. In the study by Al-Rawi et al. [95], UA concentrations in NWS and serum were significantly higher in stroke patients than in healthy controls. Serum UA did not differ significantly between stroke and stroke-risk groups, whereas NWS UA levels may serve as a predictive marker of cerebral ischemia in patients with hypertension or diabetes [95]. Similar findings have been reported in other studies [13,69]. Gerreth et al. [13] observed elevated UA in both NWS and SWS, suggesting an adaptive response to increased ROS production, while Maciejczyk et al. [69] proposed possible diffusion of UA from blood to saliva or the influence of environmental factors. While Al-Rawi et al. [95] observed no significant differences in salivary glutathione levels despite reduced serum GSH concentrations in stroke and stroke-risk patients, Gerreth et al. [13] demonstrated significantly lower GSH levels in SWS of stroke patients. Furthermore, SWS GSH concentrations were positively correlated with cognitive status (ACE-III) and dynamic balance performance (BBS). Reduced glutathione is the principal source of thiols in saliva, suggesting increased utilization in patients with stroke. The discrepancies in outcomes regarding salivary GSH may be explained by methodological differences between the studies, including differences in the stroke phase, saliva type analyzed, patient characteristics, and study design.

In both studies redox biomarkers were investigated using small case-control cohorts, nevertheless they differed substantially in their methodology. Al-Rawi et al. [95] included patients with recent ischemic stroke, a stroke-risk group, and healthy controls, whereas Gerreth et al. [13] evaluated patients in the subacute phase after both ischemic and hemorrhagic stroke and compared with healthy controls. A major strength of the study by Al-Rawi et al. [95] was the simultaneous assessment of saliva and serum, however no direct comparison between the two biological fluids was performed. In contrast, Gerreth et al. [13] focused exclusively on salivary biomarkers but implemented more detailed methodology. The inclusion and exclusion criteria were more comprehensive, smokers and patients taking vitamin supplements were excluded. Furthermore, Gerreth et al. [13] evaluated cognitive and functional status using validated clinical scales (ACE-III, BI, Functional Independence Measure (FIM), and BBS), enabling assessment of associations between salivary biomarkers and neurological outcomes, whereas Al-Rawi et al. [95] focused primarily on the diagnostic performance of the analyzed biomarkers. Although Al-Rawi et al. [95] excluded participants with active oral inflammation, advanced periodontitis, and severe gingivitis, no comprehensive assessment of oral health or salivary gland function was performed. In contrast, Gerreth et al. [13] conducted a detailed dental and periodontal examination, assessed salivary gland function, and matched the control group for oral health status, reducing the potential influence of oral conditions on salivary biomarker levels.

Malondialdehyde (MDA), a marker of lipid peroxidation, was elevated in both serum and NWS in patients with stroke and in individuals with stroke-related diseases compared with healthy controls, suggesting that salivary MDA reflects systemic oxidative stress [95]. This marker may aid in distinguishing stroke patients with hypertension or ischemic heart disease [95]. In a separate study by Al-Rawi et al. [96], salivary MDA concentrations were also significantly higher in patients with stroke. MDA levels correlated positively with low-density lipoprotein cholesterol (LDL-C) and triacylglycerols (TAGs) in both serum and saliva, and inversely with high-density lipoprotein cholesterol (HDL-C) [96]. The negative association with HDL-C is consistent with reduced atheroprotective activity. The author calculated atherogenic indices based on serum and salivary lipid ratios (LDL-C/HDL-C, total cholesterol [TC]/HDL-C, triglycerides [TG]/HDL-C), all of which were significantly higher in stroke survivors than in controls. To improve predictive accuracy, these lipid ratios were multiplied by MDA to generate a composite index. This index was significantly higher in stroke patients compared with controls, suggesting greater sensitivity for assessing cardiovascular or cerebrovascular risk [96].

## 3. Pro-Inflammatory and Anti-Inflammatory Cytokines

Cytokines and chemokines play a central role in stroke pathogenesis, driving inflammatory and prothrombotic pathways, promoting leukocyte migration, and exacerbating oxidative stress, which collectively increase infarct size and worsen neurological outcomes [111,112,113]. Consequently, inflammatory biomarkers have been proposed for stroke diagnosis. Maciejczyk et al. [12] analyzed the pro- and anti-inflammatory cytokines (TNF-α, IL-6, and IL-10) in the saliva of ischemic stroke survivors, collecting both NWS and SWS samples. Compared with healthy controls, stroke patients exhibited increased TNF-α and IL-6 levels and reduced IL-10 concentrations. According to the ROC analysis, in SWS, IL-6 concentration, TNF-α specific amount, IL-10 output, and the IL-6/IL-10 and TNF-α/IL-10 ratios achieved 100% sensitivity and specificity, while salivary TNF-α was identified as a promising biomarker for distinguishing stroke patients from healthy controls and assessing functional and cognitive impairment [12]. Forszt et al. conducted two studies evaluating a broad panel of inflammatory markers in NWS from 22 stroke patients and 22 healthy controls [114,115]. The panel included pro-inflammatory, anti-inflammatory, Th1, Th2 and Th17 cytokines, chemokines and growth factors. Compared with healthy controls, stroke patients exhibited significantly higher levels of pro-inflammatory (IL-1β, TNF-α, TNF-β), anti-inflammatory (IL-1ra, TRAIL), Th1 (IFN-γ, IL-2Rα, and IL-12 (p40)) and Th2 cytokines (IL-6), alongside reduced levels of IL-1α, IL-4 and IL-9. Among the assessed markers, TNF-α, TNF-β, IFN-γ and IL-12 demonstrated high diagnostic accuracy, achieving an AUC of 1 with 100% sensitivity and specificity. Although the results support the role of inflammation in ischemic stroke, no single immune response branch showed clear predominance [114]. The second study focused on salivary chemokines and growth factors [115]. The primary function of chemokines is to mediate cell migration, particularly of leukocytes [116]. However, their biological activity extends beyond chemotaxis, as chemokines also modulate leukocyte proliferation, survival, differentiation, cytokine production, degranulation, and respiratory burst [116]. Additionally, some chemokines influence angiogenesis and apoptosis, underscoring their broader biological roles [117]. Growth factors, in turn, regulate cell proliferation and differentiation and exert anti-inflammatory and anti-apoptotic effects that may improve outcomes after stroke [118]. The salivary analysis revealed significantly increased levels of chemokines, including CTACK/CCL27, IL-8/CXCL8, MIG/CXCL9, MIF as well as growth factors such as basic FGF, G-CSF, HGF, LIF, VEGF, in the stroke group compared with controls. The levels of MCP-3/CCL7, eotaxin/CCL11, IP-10/CXCL10, IL-3/MCGF, and PDGF-BB were significantly decreased in the study group [115].

**Figure 1:**
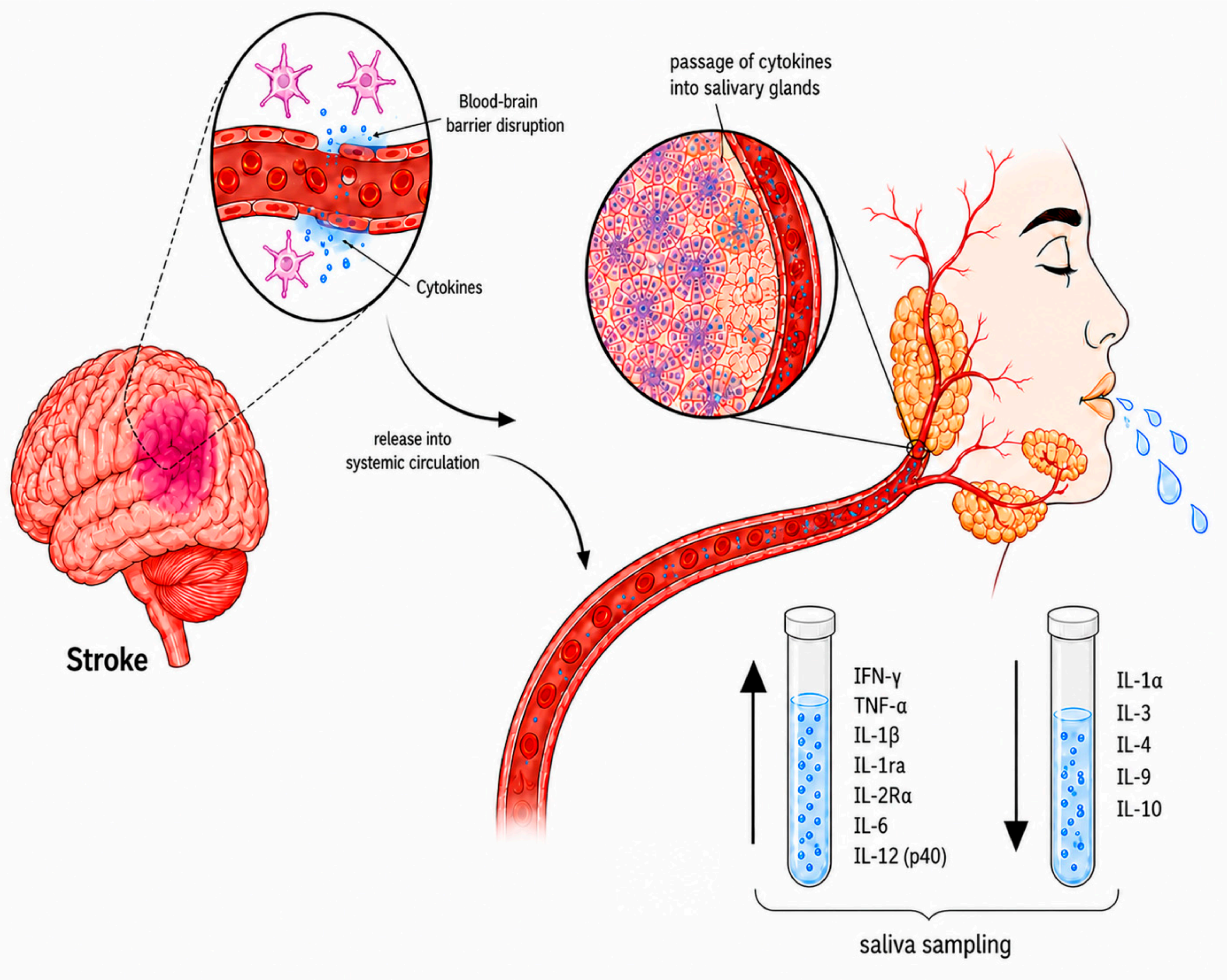
Schematic representation of the proposed pathway linking stroke-associated inflammation with the presence of inflammatory cytokines in saliva. IFN-γ—Interferon gamma; IL-1α—Interleukin 1 alpha; IL-1β—Interleukin 1 beta; IL-1ra—Interleukin 1 receptor antagonist; IL-2Rα—Interleukin 2 receptor alpha; IL-3—Interleukin 3; IL-4—Interleukin 4; IL-6—Interleukin 6; IL-9—Interleukin 9; IL-10—Interleukin 10; IL-12 p40—Interleukin 12 p40 subunit; TNF-α—Tumor necrosis factor alpha.

A notable strength of these studies was the inclusion of a control group with a comparable dental and periodontal status, minimizing the potential influence of oral health on salivary biomarker levels. In addition, smokers were excluded from the study, reducing the impact of smoking-related inflammatory changes on the results. Several cytokines correlated with functional (ADL, BBS, FIM) and cognitive (Addenbrooke’s Cognitive Examination Revised (ACE-R)) outcomes. These findings underscore associations between salivary inflammatory markers and functional outcomes, suggesting that the inflammatory profile may reflect the degree of functional impairment in stroke patients. On the other hand, these studies included a relatively small cohort and was limited to the subacute phase of stroke. Therefore, larger studies, particularly involving patients in the acute phase, are required to validate these findings and determine their clinical utility across different stroke subtypes. Future studies should also take comorbidities into consideration, as they may influence salivary inflammatory markers (Figure 1).

IL-6 and CRP levels were also measured in gingival crevicular fluid (GCF) [119], a complex fluid composed of serum exudate, tissue breakdown and turnover products, and inflammatory mediators [120]. GCF is enriched with biomarkers derived from the attachment apparatus and connective tissue [121] and contains cellular and molecular immune components that help prevent bacterial invasion from subgingival plaque [122]. GCF, present in microliter quantities in the gingival sulcus and periodontal pocket, increases in volume under pathological conditions of the gingiva and periodontium [120]. Saliva, a complex mixture, contains GCF as one of its components [123]. While salivary composition is influenced by both oral and systemic conditions, GCF profile primarily reflects the subgingival environment [124]. Although gingival crevicular fluid (GCF) is distinct from whole saliva, it is an oral fluid that contributes to the oral environment and may be present in whole saliva. We considered its inclusion valuable to provide a more comprehensive overview of oral fluid-based biomarkers. In both studies, GCF was collected by inserting paper points into the gingival sulcus for 30 s. Malik et al. performed oral examinations, including Decayed, Missing, Filled Teeth (DMFT) and PlI scores, and assessed functional and cognitive status using Modified Barthel Index (MBI) and Mini–Mental State Examination (MMSE) [58]. MMSE is a brief 30-item screening tool evaluating Attention and Calculation, Memory, Orientation, Registration, and Language, with lower scores indicating poorer cognitive function [125]. In stroke patients, mean GCF levels were 0.95 pg/mL for IL-6 and 2.62 mg/mL for CRP; no control group was included [58]. Statistical analysis showed that GCF IL-6 levels were positively correlated with dysphagia and right hemiparesis, potentially reflecting the impact of systemic inflammation on dysphagia. CRP levels in GCF were significantly correlated with IL-6, positively associated with dental plaque, and negatively related to functional dependency [58]. These findings suggest that GCF CRP levels are influenced by oral health, as CRP is produced by the gingival epithelium, and therefore may not serve as a prognostic marker for stroke outcomes [58]. In this study, the sample size (53 participants) was moderate. Moreover, oral health status as well as functional and cognitive status were evaluated. Although both ischemic and hemorrhagic stroke patients were included, the results were not analyzed separately according to stroke subtype, limiting conclusions regarding potential differences between ischemic and hemorrhagic stroke. The lack of a healthy control group makes the findings more difficult to interpret. In addition, the inclusion and exclusion criteria were not very extensive, smokers were included, and the presence of comorbidities may also have influenced the salivary biomarker levels. The authors also pointed out that medications taken by the participants could have affected the results.

Haba et al. [119] investigated serum and GCF CRP and IL-6 concentrations across different populations. Mean GCF CRP levels were 46.99 ± 232.69 mg/L in patients with transient ischemic attack (TIA) and periodontitis, 36.94 ± 224.76 mg/L in periodontitis-only participants and 1.89 ± 8.22 mg/L in healthy controls. Mean GCF IL-6 levels were 7.79 ± 22.62 pg/mL, 9.78 ± 29.71 pg/mL and 3.42 ± 2.3 pg/mL in the same respective groups. In both the periodontitis and TIA-with-periodontitis groups, significant correlations were observed between serum CRP and serum IL-6 concentrations, between serum CRP level and GCF IL-6 levels, and between serum and GCF IL-6 concentrations. Notably, serum CRP levels were significantly elevated only in individuals with periodontitis and TIA. In the periodontitis group, the correlation between serum CRP and GCF IL-6 levels was weakly negative, whereas the correlation between serum and GCF IL-6 was positive. This discrepancy suggests that in periodontitis, local IL-6 concentrations may influence systemic levels of this marker, whereas CRP concentrations do not reflect periodontal status, consistent with the lack of correlation between local and systemic CRP levels. A score function was applied to evaluate the probability of cerebrovascular disease in patients with periodontal disease based on specific factors. Among the parameters assessed, bleeding index, bone loss, GCF collection depth, and periodontal disease status reached statistical significance. The researchers concluded that TIA development could be predicted solely by serum IL-6, indicating that local inflammatory markers are not linked to TIA occurrence [119]. The comparison included patients with transient ischemic attack (TIA) and periodontitis, patients with periodontitis, and healthy controls, making it possible to evaluate the influence of periodontal disease on GCF biomarkers. However, the study did not compare stroke with other neurological conditions. Furthermore, the inclusion and exclusion criteria were not clearly described, and no information on smoking status or medication use was provided, although both factors could have influenced the biomarker levels.

The study of Pawlukowska et al. [126] aimed to evaluate the impact of periodontal disease and salivary stimulation on the clinical course of acute ischemic stroke. In addition, the authors investigated whether salivary inflammatory biomarkers were associated with periodontal status and stroke severity. Salivary IL-1β, MMP-8, OPG and RANKL concentrations in patients with first ischemic stroke undergoing different treatment approaches were assessed. Neurological deficits were assessed on hospital days 1, 3, and 7 using the National Institute of Health Stroke Scale (NIHSS). Oral examinations evaluated missing, carious and filled teeth, as well as dental deposits, periodontal pocket depth, bleeding on probing, and tooth mobility. In the study population, 36% of participants had periodontal disease. Baseline NIHSS scores indicated more severe neurological deficits in patients with periodontal pathology. Additionally, these patients exhibited a higher frequency of respiratory tract infections. NIHSS scores on days 3 and 7 showed greater neurological improvement in patients without periodontal disease. Interestingly, no significant differences were observed in IL-1β, MMP-8, OPG or RANKL concentrations in NWS between stroke patients who underwent salivary gland stimulation and oral hygiene care and those who did not [126].

Palm et al. [127] designed a study investigating a panel of biomarkers similar to that evaluated by Pawlukowska et al. [126], however, their objective was to determine whether exposure to salivary periodontal pathogens was associated with the risk of acute ischemic stroke. Researchers included both a stroke group and a control group of healthy individuals. Participants provided information on stroke risk factors, including hypertension, diabetes mellitus, hypercholesterolemia, atrial fibrillation, coronary artery disease, peripheral artery disease, smoking status, BMI, number of remaining teeth and history of periodontitis; no oral examination was performed. Serum and SWS samples were collected within 24 h of admission. Subjects in the stroke group more frequently presented with risk factors and were edentulous or had fever teeth than controls. After adjusting for stroke risk factors and number of remaining teeth, salivary and blood IL-1β, MMP-8, MPO concentrations, as well as serum lipopolysaccharide (LPS) activity and A. actinomycetemcomitans levels, were significantly higher in the SWS of the controls compared with the stroke group. Conversely, serum MPO and MMP-8 levels were markedly elevated in stroke survivors relative to healthy individuals. PCR testing showed that stroke survivors were more frequently carriers of A. actinomycetemcomitans. Moderate correlations were observed between salivary IL-1β, MMP-8, MPO levels, TIMP-1 ratio, and number of remaining teeth [127], suggesting that controls exhibited active periodontitis with tissue destruction and local inflammation, whereas stroke patients had fewer or no teeth, reflecting the final stage of periodontal disease leading to tooth loss. Given that diagnostic methods, sample sizes and type of stroke were similar, this findings may suggest that the type of saliva analyzed affects the detection of IL-1β and MMP-8. Moreover, Pawlukowska et al. [126] did not report smoking status or medication use, despite both factors potentially influencing periodontal status, inflammatory marker levels, and stroke outcomes. In contrast, Palm et al. [127] adjusted their analyses for smoking and major risk factors; however, the inclusion and exclusion criteria remained relatively broad. Consequently, the inclusion of patients with conditions potentially affecting salivary biomarkers were not excluded, increasing the risk of confounding. Conversely, Pawlukowska et al. [126] excluded patients with disorders known to impair salivary secretion.

Al-Rawi et al. [128] investigated salivary neuron-specific enolase (NSE), a molecule that promotes pro-inflammatory cytokine production [129], induces ECM degradation [130], and may contribute to neuronal apoptosis [131]. Beyond its neuroinflammatory role, NSE regulates neuronal differentiation, survival, growth, and neuronal death [132], and is considered a reliable biomarker of neuronal injury [133]. Numerous studies have demonstrated significant correlations between serum NSE levels and infarct volume in ischemic stroke [134,135], degree of disability [136,137], neurological outcome [131,133,134] and severity of neurological deficits [135]. On this basis, Al-Rawi et al. [128] evaluated the utility of NSE as a diagnostic and monitoring biomarker in ischemic stroke and as a predictive marker in individuals with stroke risk factors. They found that salivary NSE concentrations were significantly elevated in both post-stroke patients and individuals with stroke risk factors, potentially reflecting varying degrees of BBB disruption and subsequent enzyme translocation into blood and saliva. Serum NSE levels were significantly higher in stroke patients than in controls; however, among risk groups, elevated serum NSE was observed only in patients with diabetes. These findings suggest that while serum NSE may aid in distinguishing stroke patients from healthy individuals, its predictive utility remains limited. The authors emphasized its potential role in identifying stroke patients and estimating stroke risk [128]. An important strength of this study was the inclusion, in addition to stroke patients and healthy controls, of a separate group of patients with stroke-related risk factors, providing a more clinically relevant comparison. The authors also took oral health status into account by excluding patients with oral inflammation. Another advantage was measuring NSE in both saliva and serum as salivary biomarkers may reflect systemic changes. However, no comparison between salivary and serum outcomes was performed, no information was provided regarding the stroke phase at the time of sample collection, and stroke severity was not assessed, making the interpretation of NSE levels more difficult.

## 4. Cortisol

Cortisol is a glucocorticoid synthesized in the zona fasciculata of the adrenal cortex [138]. Its secretion is regulated by the hypothalamic-pituitary-adrenal (HPA) axis. Cortisol exerts pleiotropic effects, including anti-inflammatory activity and maintenance of energy homeostasis through stimulation of gluconeogenesis, thereby supporting the metabolic demands of vital organs, particularly the brain [139]. In patients with stroke, a sustained activation of the HPA axis may occur secondary to systemic inflammation, cytokine release, or disruption of central inhibitory pathways [65]. Consequently, elevated cortisol levels may represent a direct neuroendocrine response to acute stroke. Saliva is considered the preferred specimen for cortisol assessment, as it reflects the unbound, biologically active fraction of the hormone, in contrast to serum measurements, which include protein-bound cortisol.

Several studies have evaluated salivary cortisol in stroke patients in relation to clinical parameters. Ahmed et al. [140] examined associations between cortisol and blood pressure (BP), systolic (SBP) and diastolic (DBP), in patients with ischemic stroke. Elevated salivary cortisol levels were associated with a higher 24-h mean BP and heart rate (HR). The 24-h mean salivary cortisol correlated positively with 24-h mean SBP, day-time SBP, night-time SBP, and nighttime DBP. Morning salivary cortisol correlated positively with nighttime SBP, whereas afternoon measurements showed no significant associations with BP parameters. A major strength of this study was the assessment of patients in the acute phase of ischemic stroke, including stroke severity via NIHSS evaluation, repeated saliva sampling over a 24-h period, and continuous blood pressure monitoring. The authors also accounted for important vascular risk factors and antihypertensive treatment in their analyses, reducing the influence of potential founders. Nevertheless, moderate sample size, and inclusion of ischemic stroke patients only limit the universality of the findings. The tests were performed on average about 10 h after the stroke, which also affects the potential of the results. Although reference cortisol values from a healthy working population were used, no direct comparison with a matched control group was performed. Furthermore, the study was not to evaluate the diagnostic performance of salivary cortisol designed in differentiating stroke from other neurological conditions. Laures-Gore et al. [141,142] conducted two studies, which addressed different research questions. The first one focused on cortisol reactivity to experimentally induced linguistic stress and included a healthy control group [141], whereas the second investigated longitudinal changes in salivary cortisol during recovery by comparing patients with left- and right-hemisphere stroke [142]. In the first one, cortisol levels were compared between healthy controls and patients with left-hemispheric stroke and aphasia. Participants underwent the Trier Social Stress Test (TSST) and a non-linguistic stress task, the Mirror Drawing Test (MDT). NWS samples were collected during a 30-min baseline period, at the beginning and end of each task, and at 10-min intervals for 90 min post-task. Cortisol levels increased after task completion in both groups, however overall cortisol concentrations were higher in the stroke group than in controls [141]. In a subsequent study by the same authors, only post-stroke patients were included, categorized according to lesion laterality: left hemisphere (LH) versus right hemisphere (RH). Saliva samples were collected biweekly for 3 months. Cortisol levels did not differ significantly between the groups over time. However, in the RH group, cortisol levels were negatively correlated with naming ability on the Boston Naming Test [142]. The effect of the TSST on cortisol dynamics after ischemic stroke was also investigated by Mirzaee et al. [143] NWS samples were collected at four time points: pre-TSST, immediately post-TSST and 20 and 40 min after task completion. At baseline, patients with ischemic stroke exhibited significantly higher salivary cortisol levels than controls. No significant post-stress cortisol changes were observed in the stroke group [143]. In the case of the three aforementioned studies, there are similarities and differences that may translate into their strength or limitations. The first study of Laures-Gore et al. included both stroke patients and healthy controls, allowing direct comparison between groups. In addition to salivary cortisol measurements, participants also provided subjective ratings of perceived stress following the experimental task. However, the study included only patients with left-hemisphere stroke and aphasia, and no information on smoking status was provided [144]. The second research was designed as a prospective longitudinal study, enabling the assessment of changes in salivary cortisol during recovery after stroke [145]. In contrast, Mirzaee et al. [143] investigated only patients with chronic ischemic stroke and used the Trier Social Stress Test (TSST) to induce stress. Besides salivary cortisol, the authors also evaluated blood pressure, heart rate and serum oxidative stress markers such as MDA, CAT and glutathione, providing a more comprehensive assessment of the stress response [143]. A common strength of all three studies was the repeated collection of saliva samples, which increases the reliability of cortisol assessment and allows evaluation of its dynamic changes rather than relying on a single measurement. Nevertheless, all studies were limited by relatively small sample sizes. In addition, neither study by Laures-Gore specified the ischemic stroke subtype, whereas Mirzaee et al. [143] included only patients with ischemic stroke. All three studies also attempted to standardize saliva collection by restricting factors known to influence cortisol secretion. In the studies by Laures-Gore et al. [141,142], participants were instructed to refrain from smoking, consuming caffeine, alcohol, or food for two hours before saliva collection, whereas Mirzaee et al. [143] required participants to avoid smoking for at least one day and food for one hour before testing.

Wang et al. [146] compared salivary cortisol concentrations in patients with cerebral ischemic stroke with mild cognitive impairment (CIS-MCI) and without cognitive impairment (CIS). The aim of the study was to investigate whether salivary cortisol predicts mild cognitive impairment after ischemic stroke. Samples were obtained at 8 a.m. on the morning following enrollment. Salivary cortisol levels were significantly higher in the NWS of the CIS-MCI group than in the CIS group. Higher cortisol concentrations were also associated with elevated LDL-C and TC levels, current or past hyperlipidemia, and advanced age [146]. Collectively, these findings suggest that elevated cortisol levels are associated with greater post-stroke impairment and may reflect overall disease severity.

Moreover, Tene et al. [144] investigated the association between post-stroke cortisol levels, cognitive decline, and structural brain abnormalities. Salivary cortisol was measured in 182 patients with ischemic stroke or TIA within 72 h of admission. Samples were collected in the evening and at 7:30 am after admission (approximately 30 min after awakening), and at 6, 12 and 24 months following stroke onset. Patients were stratified into tertiles according to bedtime cortisol levels. Elevated bedtime salivary cortisol was significantly associated with a greater neurological deficit, higher white matter lesion burden, thinner parietal cortex, reduced white matter volume, and poorer global microstructural integrity of the white matter and hippocampus. No association was observed with ischemic lesion volume or total intracranial volume. Morning cortisol concentrations were not associated with neuroimaging abnormalities. Baseline salivary cortisol levels were elevated in all patients compared with follow-up measurements at 6, 12, 24 months. In the high cortisol subgroup rates remained higher than rates from the low cortisol subgroup at 6, 12, and 24 months after stroke. At admission, patients in the high cortisol subgroup had significantly lower total white and gray matter volumes, smaller hippocampal volumes, greater white matter lesion volume, and poorer global white matter microstructural integrity compared with the low cortisol subgroup. Cognitive status was assessed using multiple instruments, including the Montreal Cognitive Assessment (MoCA), a screening tool for Mild Cognitive Impairment (MCI), a condition associated with increased risk of dementia [145]. The MoCA evaluates short-term memory recall, visuospatial abilities, executive function, attention, concentration, working memory, language, and orientation [145]. Cognitive decline scores were not associated with morning cortisol concentrations. In contrast, higher bedtime cortisol levels were significantly correlated with poorer executive function and attention scores at admission and at 6, 12, and 24 months, as well as with lower global computerized cognitive scores at 12 and 24 months after stroke. Significant differences in executive function and memory were observed between cortisol subgroups. Patients in the low cortisol subgroup demonstrated sustained improvement at 24 months, whereas those in the high cortisol subgroup exhibited progressive cognitive decline. Higher bedtime cortisol levels were additionally associated with poorer visuospatial function at 12 months and with lower global cognitive scores, impaired motor function and worse memory at 24 months post-stroke. The authors suggested that neuroimaging abnormalities observed in patients with elevated bedtime cortisol may represent pre-existing brain atrophy predating the stroke or TIA. Reduced global white matter microstructural integrity in conjunction with elevated cortisol levels may indicate stress-related neurodegenerative processes. Acute vascular events constitute a significant physiological stressor that may modulate HPA axis activity. The authors proposed that HPA axis dysregulation is not merely a consequence of stroke or TIA but may represent a pre-existing characteristic of these patients, potentially reflecting diminished capacity to cope with acute vascular events and thereby contributing to poorer cognitive outcomes. This vulnerability may also be linked to structural brain atrophy. Acute cortisol measurements appeared most informative, as cortisol levels during follow-up visits showed limited associations with cognitive and imaging parameters. Disruption of the circadian rhythm of HPA axis activity may therefore serve as a predictor of subsequent cognitive impairment [144].

The study by Zhanina et al. [147] included 45 patients with stroke and 32 healthy controls. The objective was to evaluate corticoid-dependent and sympathetic-related mechanisms underlying depressive symptoms and cognitive impairment. Salivary cortisol and α-amylase were measured during the acute stroke phase and at 30, 180 and 365 days after stroke. In the acute phase, α-amylase concentrations were lower in the stroke group than in controls. A significant increase in α-amylase was subsequently observed in the stroke group at 30, 180 and 365 days compared with the acute phase. Cortisol levels were significantly higher in the stroke group than in controls at 30, 180 and 365 days after stroke, with the highest levels observed at 1 year. Salivary cortisol at 365 days was significantly elevated compared with the acute phase. α-Amylase levels did not differ between patients with and without cognitive impairment. In contrast, cortisol concentrations in patients with cognitive decline were higher in the acute phase than in patients without cognitive impairment, suggesting an association between cognitive dysfunction and HPA hyperactivation. However, cortisol levels did not differ significantly between patients with and without depressive disorder. α-Amylase was markedly increased in patients with depressive disorder and remained elevated at 365 days after stroke. In patients without depressive disorder, α-amylase was elevated only up to 180 days after stroke, and returned to baseline within a year. These findings suggest that prolonged sympathetic nervous system hyperactivation, reflected by sustained elevation of α-amylase, may represent a stress response to stroke [147]. Salivary α-amylase is not a component of the hypothalamic-pituitary-adrenal axis; rather, its activity reflects sympathetic nervous system activation and increases in response to physical or psychological stressors [148].

Kwon et al. [149] evaluated the association between cortisol levels and depressive status in 28 patients with poststroke depression and 23 healthy caregivers. Morning salivary cortisol was collected using the Salivette immediately upon awakening and at 15, 30 and 45 min thereafter, over two consecutive days. In the study group, no significant increase in cortisol concentration was observed at any sampling time. In contrast, the control group demonstrated a significant rise at 15 and 30 min after awakening compared with baseline. Although cortisol levels at awakening did not differ between groups, significant differences were observed at 15, 30 and 45 min after awakening. Cortisol levels were significantly higher in controls than in patients at 15 and 30 min after awakening. Both the early-morning cortisol increase and total cortisol output at 45 min were significantly lower in the study group. In contrast to previous findings, cortisol levels in the study group were negatively correlated with depression severity as assessed by the Korean version of the Beck Depression Inventory II (BDI) and the Hamilton Depression Rating Scale (HDRS). These findings support HPA axis dysregulation in post stroke depression. Lueken et al. investigated the impact of stroke laterality on stress responses [150]. 32 patients with stroke, categorized into LH (*n* = 18) and RH (*n* = 14) subgroups, and 30 healthy controls were included. Basal cortisol levels in NWS were measured on 3 consecutive mornings. Phasic cortisol responses were assessed by NWS collection twice before a cognitively demanding task, immediately after task completion, and at 10-min intervals for 1 h. No significant differences in basal cortisol levels were observed between the stroke group and controls; however, basal cortisol was significantly higher in the LH subgroup compared with controls. No significant differences were found between LH and RH subgroups. In phasic measurements, cortisol levels in the stroke group increased significantly at T7 (i.e., 40 min after task completion) compared with the practical baseline (PB); similar findings were observed in the LH subgroup. Higher cortisol values were noted in the RH subgroup than in the LH subgroup at the second baseline measurement (T2), although this difference did not reach statistical significance. Lower cortisol levels were associated with more anterior lesion location, particularly in the RH subgroup. These findings suggest that stroke may disrupt both basal and stress-induced HPA axis activity. The affected hemisphere and intrahemispheric lesion location may influence stress regulation, with the right hemisphere playing a dominant role in HPA axis regulation and the left-hemisphere contribution to the temporal regulation of stress responses.

Several methodological differences between these studies should be considered when interpreting their findings. Wang et al. [146] and Tene et al. [144] included relatively large study populations, although neither study incorporated a healthy control group. In contrast, Zhanina et al. [147] and Kwon et al. [149] included healthy controls, but both studies utilized relatively small sample sizes. Except for Zhanina et al. [147], who did not specify the type of saliva collected, the remaining studies analyzed NWS. Repeated saliva sampling was performed by Tene et al., Zhanina et al. [147], and Kwon et al. [149], allowing assessment of temporal changes in cortisol levels, whereas Wang et al. [146] relied on a single saliva sample. Although none of the studies performed a comprehensive dental or periodontal examination, Zhanina et al. [147] reported that none of the participants presented inflammatory or other visible lesions in the oral cavity. Similarly, Wang et al. [146] confirmed the absence of oral ulcerations during saliva collection to minimize sample contamination. In addition, both Tene et al. [144] and Kwon et al. [149] implemented saliva collection protocols to minimize blood contamination of the samples. Nevertheless, the lack of a standardized assessment of periodontal status remains an important methodological limitation. Furthermore, Wang et al. [146], Tene et al. [144], and Zhanina et al. [147] included only patients with ischemic stroke, whereas Kwon et al. [149] did not specify the stroke subtype. Consequently, the applicability of these findings to hemorrhagic stroke and different ischemic stroke subtypes remains uncertain.

The objective of the study of Atam et al. [151] was to investigate the association between circadian rhythm biomarkers and stroke severity and functional outcomes in patients with acute ischemic stroke. It was stated that salivary cortisol levels were higher in patients with stroke than in controls, although the difference did not reach statistical significance [151]. Stroke severity was assessed using the NIHSS at admission, while functional outcome was evaluated with the modified Rankin Scale (mRS) at admission and discharge. Cortisol levels were significantly associated with both stroke severity and functional status. Higher salivary cortisol concentrations were observed in patients with better functional outcomes according to the mRS; however, cortisol levels were also positively associated with stroke severity. The authors suggested that elevated cortisol reflects neuroendocrine stress responses in more severe strokes and may be linked to poorer clinical outcomes [151]. The main strengths of this study include the incorporation of a healthy control group, allowing direct comparison of biomarker levels. Additionally, simultaneous assessment of several circadian rhythm parameters, including salivary cortisol, urinary melatonin, and 24-h ambulatory blood pressure monitoring (ABPM), provides a more comprehensive evaluation of circadian rhythm disturbances following stroke. However, several limitations should also be acknowledged. The study included a moderate sample size and was restricted to patients with ischemic stroke, limiting the universality of the findings to other stroke subtypes, particularly hemorrhagic stroke. Furthermore, salivary cortisol and urinary melatonin were each measured at a single time point, limiting assessment of their dynamic circadian fluctuations. The authors also did not account for important confounding factors, including sleep quality, psychological stress, medication use (e.g., corticosteroids, β-blockers, and hypnotics), frailty, and light exposure, all of which may influence cortisol and melatonin secretion. The lack of long-term follow-up limited the evaluation of the prognostic value of these biomarkers for long-term functional recovery, cognitive decline, and recurrent stroke. Finally, interpretation is limited by insufficient methodological details regarding saliva collection, statistical analysis, and assay techniques. Without oral and periodontal assessment the procedure of saliva sampling enables potential contamination of saliva samples with blood.

## 5. Oral Microbiota

Communication pathways such as the oral-gut axis and the gut-brain axis have recently been investigated in stroke [152]. The gut-brain axis contributes to maintaining central homeostasis and modulates immune and inflammatory responses [153]. This bidirectional signaling influences neurogenesis, neurotransmission, neuroinflammation and stress responses [154]. Evidence suggests that oral *Streptococcus* species may contribute to cerebrovascular events. DNA of viridans streptococci has been detected in thrombi aspirated from patients with ischemic stroke [155]. In addition, *Streptococcus mutans* strains expressing collagen-binding protein have been implicated as a potential risk factor for hemorrhagic stroke [156]. Bacteremia following dental procedures may allow these bacteria to enter the bloodstream and bind to exposed collagen at sites of endothelial injury. This interaction may inhibit collagen-induced platelet aggregation, and promote MMP-9 activation, thereby increasing the risk of bleeding [156].

Several studies on post-stroke populations focused on microbiological analyses. In one study by Cieplik et al. [157], samples were collected from the dorsal tongue and subgingival plaque/alveolar ridges to assess the composition of the oral bacterial biofilm. Sampling was performed at three time points: within 24 h of admission (baseline), and at 48 and 120 thereafter. Patients were divided into 3 subgroups: those with uncomplicated stroke, those who developed post-stroke pneumonia and a control group consisting of individuals with stroke mimics. Overall, no significant changes in microbial composition were observed [157]. In another study, the collagen-binding protein (CNM) expressed by *Streptococcus mutans*, one of the most prevalent cariogenic pathogens, was significantly elevated in patients with neural injuries, including intracerebral hemorrhage [158]. Interestingly, Chen et al. [152] demonstrated that mice subjected to gavage with saliva from individuals with periodontitis following middle cerebral artery occlusion had significantly worse outcomes than those receiving saliva from healthy controls. This was associated with enhanced neuroinflammation, characterized by increased infiltration of inflammatory cells into brain tissue and elevated expression of proinflammatory mediators, including IL-1β, TNF-α, Th17 cells and IL-17+ γδ T cells (IL-17A-producing cells). In contrast, mice receiving saliva from healthy individuals did not exhibit increased Th17 or IL-17+ γδ T cell infiltration in post-stroke brain tissue [152].

The oral microbiome was also assessed by Manzoor et al. [159], who enrolled 155 patients with cryptogenic ischemic stroke (CIS) and 153 healthy controls. Microbiological evaluation was conducted using SWS samples. Overall microbial community structure did not differ significantly between groups. The salivary microbiome was predominantly composed of the phyla Bacillota, Actinomycetota, Bacteroidota, Pseudomonadota and Fusobacteriota. However, the study group demonstrated a significantly higher relative abundance of *Brenneria goodwinii*, *Variovorax boronicumulans*, *Pseudomonas* sp. *AN-B15*, *Actinoalloteichus* sp. *GBA129-24* and *Thiomonas arsenitoxydans* compared with controls, and a significantly lower abundance of *Metamycoplasma alkalescens*, *Streptomyces* sp. *LBUM 1475*, *Pseudonocardia dioxanivorans*, *Arthrobacter dokdonella* and *Francisella frigiditurris*. These bacterial species may be associated with pathological processes occurring before or after CIS. Notably, certain viral, fungal, and archaeal communities also differed significantly between groups, including *Brussowvirus ALQ132* and *Moineauvirus Abc2* as well as *Fusarium venenatum*, however, due to limited data, their functional relevance remains unclear. Significant enhancement of inosine 5′-phosphate biosynthesis II and III, along with stachyose degradation pathways, was reported in the study group. Inosine pathways are involved in purine metabolism and extracellular inflammatory signalling, whereas stachyose metabolism may promote bacterial growth in the gut. These findings suggest a potential contribution of the salivary microbiome to oral-gut-brain axis communication. Increased network complexity and species clustering further support the need for additional investigation [159].

The same research group conducted another study evaluating SWS microbiology in patients with cryptogenic ischemic stroke [160], divided into two equal subgroups: those with high-risk PFO and those without PFO. A control group of stroke-free individuals with high-risk PFO was also included. Overall salivary microbiota distribution was consistent with prior findings. No significant differences were observed between high-risk and no-risk PFO stroke subgroups, nor between stroke patients with high-risk PFO and stroke-free controls with high-risk PFO. However, the high-risk PFO stroke subgroup demonstrated significantly increased abundance of Ascomycota and Saccharomycetes compared with the no-PFO stroke subgroup. Additionally, compared with stroke-free individuals with high-risk PFO, the high-risk PFO stroke subgroup exhibited increased levels of Bacteroidota and Synergistota and decreased abundance of genus *Lactococcus*, including *Lactococcus raffinolactis* and *Lactococcus cremoris* [160]. Dysbiosis of the oral microbiome, particularly with an increased representation of Gram-negative species, may promote thrombogenesis and may be associated with PFO and PFO-related paradoxical embolic stroke mechanisms [159].

In the study by Tonomura et al. [161], the microbiome composition was consistent with previous reports and did not differ significantly between stroke and control individuals, despite the use of NWS samples for evaluation. Fecal samples were also analyzed for comparison. During this prospective study, data were collected from electronic medical records, including all-cause mortality and hospital admissions for cardiovascular events such as stroke, heart failure, and myocardial infarction. Gut microbiological diversity was notably reduced in the stroke group compared with healthy individuals. Nevertheless, *Streptococcus anginosus* was markedly enriched in both saliva and fecal samples of the stroke patients. Multivariable analysis demonstrated that *Streptococcus anginosus* in gut microbiome was independently associated with stroke and served as a prognostic marker for mortality and cardiovascular events. Furthermore, *Treponema denticola*, *Streptococcus cristatus*, *Prevotella loescheii* and *Streptococcus anginosus* were more abundant in the oral microbiome [161].

According to Roongpiboonsopit et al. [162], salivary microbiota composition differed significantly in stroke patients, with increased operational taxonomic units (OTUs) and Chao1 richness, indicating enhanced microbial diversity. The stroke group exhibited a markedly higher abundance of phylum Bacillota and *Streptococcus* species, with a reduced contribution of Bacteroidota, Actinomycetota and Pseudomonadota and *Prevotella* in the salivary microbiota. Fusobacteria showed minimal increase in stroke patients. Notably, *Streptococcus infantis* levels were elevated, while *Prevotella melaninogenica* levels were reduced, although the latter did not reach statistical significance. Elevated *Streptococcus* has been implicated in systemic inflammation and the pathogenesis of atherosclerosis [162]. Microbial functional analysis revealed substantial remodeling in stroke patients, with increased xenobiotic degradation, enhanced membrane transport, and activation of cardiovascular-related pathways, whereas controls showed greater enrichment of replication and repair, energy metabolism, and cellular processes [162]. Overall, the oral microbiome of stroke patients exhibited greater bacterial heterogeneity, including an increased presence of potentially pathogenic and opportunistic taxa, which may promote post-stroke inflammation and contribute to systemic consequences. These compositional and functional changes likely reflect microbial adaptation to the inflammatory and oxidative stress–rich environment associated with acute ischemic stroke (AIS) and transient ischemic attack (TIA) [162].

Collectively, these findings suggest that oral microbial status may contribute to stroke occurrence and influence post-stroke outcomes. However, further studies are required to elucidate the underlying mechanisms.

## 6. Other Biomarkers

Additional reports highlight salivary biomarkers, particularly brain proteins involved in stroke pathogenesis. Brain-derived neurotrophic factor (BDNF), a member of the neurotrophin family [163], is expressed in regions critical for learning and memory, including the hippocampus, cerebellum, cerebral cortex and amygdala [163]. In adulthood, BDNF promotes differentiation and development of central and peripheral neurons [164]. It is essential for regulating synaptic plasticity, neurotransmitter release, synapse structure and synaptic connectivity [165]. During stroke, BDNF exhibits neuroprotective effects, including anti-apoptotic and anti-inflammatory activities, reduction of free-radical formation, reduction of extracellular glutamate excitotoxicity, and regulation of intracellular calcium mobilization [166]. A polymorphism in the *BDNF* gene distinguishes carriers of the Met allele from individuals homozygous for the Val66Val genotype. Met allele carriers are generally associated with more severe neurological deficits than Val66Val genotype [167]. Dresang et al. [167] investigated the relationship between *BDNF* polymorphism and post-stroke aphasia using saliva samples. The study included 17 individuals with a single left-hemisphere ischemic stroke [167]. Aphasia severity was assessed with the Western Aphasia Battery Aphasia Quotient (WAB-AQ) [168]. Cortical excitability and neuroplasticity were measured via motor-evoked potentials (MEPs) before and after inhibitory continuous theta burst stimulation (cTBS). Older participants exhibited greater aphasia severity, particularly among Val66Val carriers compared to Val66Met carriers, whereas younger Val66Val carriers had less severe aphasia than their Val66Met counterparts. WAB-AQ scores correlated positively with Val66Val carriers and negatively with Val66Met carriers [167]. Higher WAB-AQ, reflecting less severe aphasia, were associated with greater MEP suppression in both genotypes, and were more pronounced in Val66Met carriers [167]. These findings suggest that salivary genetic biomarkers, combined with neurophysiological measures, can serve as indicators of neuroplasticity and may enhance prognostic assessment of aphasia severity.

Substance P (SP) is present in both the central and peripheral nervous system [169] and contributes to inflammation, thrombosis and neurogenic inflammation after stroke [170] promoting blood–brain barrier permeability and cerebral edema [171]. Several studies have linked SP to dysphagia [172,173,174], a common post-stoke complication [175]. Muhle et al. [172] reported that successful treatment of dysphagia correlated with increased salivary SP levels. Patients with low salivary SP had reduced spontaneous swallowing and a higher incidence of pneumonia [173]. Conversely, Alvarez-Larruy et al. [174] found no significant differences in SP levels in NWS between post-stroke patients with or without dysphagia.

Sun et al. [176] measured melatonin in NWS from 182 AIS patients and 73 controls. While melatonin primarily regulates circadian rhythms [177], it also functions as a free-radical scavenger and indirect antioxidant [178], neutralizing hydroxyl radicals and peroxynitrite anions and reducing lipid peroxidation [179], thereby mitigating oxygen-induced neurotoxicity [180]. Salivary melatonin was collected at six time points (0:00, 3:00, 6:00, 12:00, 18:00, 21:00) from 22 stroke patients and 10 controls. Stroke patients had significantly lower melatonin levels at all time points, reduced AUC values, diminished amplitude and peak, and delayed peak time (03:00 vs. 00:00 in controls). Hourly NWS sampling between 19:00 and 23:00 was used to determine dim light melatonin onset (DLMO; the time when salivary melatonin reached 4 pg/mL), the post-DLMO surge (melatonin secretion rate over the 30 min following DLMO), and the 30-min AUC post-DLMO. All parameters were significantly lower in stroke patients, with a 94-min DLMO delay that was statistically significant. Based on DLMO, patients were categorized as having advanced (<19:30), normal (19:30–22:00), or delayed (>22:00) circadian rhythms; 19 had advanced and 51 had delayed rhythms. Three-month neurological recovery was poor in 37 of 160 patients, who exhibited decreased melatonin at 22:00 and delayed DLMO. These findings indicate that acute stroke may disrupt central neuroendocrine regulation, alter circadian rhythms, and adversely affect short-term neurological recovery [176].

Tong et al. [180] evaluated salivary formaldehyde as a potential biomarker in stroke. Formaldehyde is a reactive cytotoxic compound that induces endothelial damage, protein glycation and ROS production [181,182,183]. The study included 346 participants divided into 3 subgroups: 61 stroke survivors, 65 with post-stroke dementia (PSD), and 220 with Alzheimer disease, alongside 231 healthy controls. Formaldehyde may be associated with vascular injury and cognitive impairments as assessed by ADL, Clinical Dementia Rating (CDR) and MMSE scores. CDR is a standardized instrument that evaluates cognitive decline through a semi-structured interview with a knowledgeable caregiver or family member. It employs a five-point scale across six domains relevant to dementia: personal care, home and leisure activities, memory, community functioning, judgment and problem-solving. Higher CDR scores reflect more severe functional and cognitive deterioration. Urine, blood and NWS samples were collected from participants. Cognitive status scores did not correlate with saliva concentrations of formaldehyde [180], highlighting an area for further investigation.

Among the enzymes commonly present in saliva, only one non-antioxidant and non-inflammatory enzyme was assessed: salivary amylase, which facilitates the pre-digestion of polysaccharides [184,185], and inhibits the growth of certain bacteria [186,187]. However, it is also a component of dental plaque [188], binding to early colonizers such as streptococci, suggesting a role in plaque formation [189,190]. In the study by Maciejczyk et al. [61], amylase activity in the NWS was significantly lower in the HS and NS subgroups compared to controls. Similarly, amylase activity in SWS was markedly reduced in the HS subgroup relative to the NS subgroup and controls. Moreover, elevated oxidative and nitrosative stress strongly correlated with salivary amylase activity, total protein content and NWS flow rate. This may reflect oxidative stress-induced salivary gland damage, which alters gland function and saliva composition [61].

Data on the analyzed biomarkers are summarized in Table S1, while the saliva collection methodology is described in Table S2.

## 7. Strengths and Limitations

The use of saliva as a diagnostic tool has been of clinical interest for many years. Saliva collection provides a non-invasive method to detect diseases, monitor their progression and evaluate the treatment efficacy [191]. Economically, saliva is an attractive biofluid compared with others [192], as collection, storage, and shipping are simpler and less expensive than for blood [62]. Collection does not disrupt skin or mucosa [193], reduces risk of pathogen exposure for healthcare professionals [194], and requires smaller sample volumes [195]. Saliva sampling also bypasses clotting considerations [196] and is generally more comfortable for patients, particularly children, the elderly, or individuals with cognitive impairments [197].

Two types of saliva should be considered: unstimulated and stimulated [198]. Unstimulated saliva can be collected via active spitting or passive drooling [199]. Stimulated saliva is produced through mastication [200], often using external stimulants such as: paraffin wax, chewing gum, citric acid, or lemon drops [199]. Participants should refrain from oral hygiene procedures, eating, drinking [200] and smoking for at least 2 h prior to sampling [201]. The mouth should be rinsed with deionized water, followed by five minutes of limited orofacial movement [201]. During collection, participants should sit with their heads slightly bowed [202]. Samples should be kept on ice to maintain protein integrity, with long-term storage at −20 °C and ideally at −80 °C [203]. Optimal collection is between 8:00 and 10:00 a.m. [200], as salivary components and flow rate follow circadian rhythms [204]. Cortisol in particular [205], peaks within 30–40 min after waking, then declines sharply before gradually tapering off until bedtime [206].

Limitations of saliva collection include potential contamination with blood due to mucosal injury or gingivitis [207]. Saliva composition also varies with collection method, salivary flow [208], body posture, hydration, nicotine usage, medications, diet, psychological state [201], and gender [209,210]. Physical exertion can alter salivary proteomics [211,212], and age-related glandular changes affect composition and flow [213,214]. In elderly patients, medications can cause degeneration of the salivary glands, leading to changes in saliva composition and reduced salivary secretion [215]. The most common drugs associated with hyposalivation include: benzodiazepines, phenothiazines, anticholinergics, antidepressants, antihistamines, proton pump inhibitors, diuretics, statins [215] opioids and antihypertensives such as alpha-1 antagonists, alpha-2 agonists, and beta blockers [216]. Additional agents, including magnesium hydroxide, ophthalmologicals, glucosamine and urinary antispasmodics are also linked to reduced salivary flow [217]. Histological changes in the salivary glands are associated with acinar degeneration [214], accompanied by fibrosis and fat deposition within the glandular tissue [213]. Moreover, depression, stress, anxiety, or fear resulting from physical and cognitive decline can further reduce salivary secretion in the elderly [216].

Salivary biomarkers are extensively analyzed in relation to systemic diseases [218], with inflammatory and oxidative stress markers found to be elevated in conditions such as diabetes [219], chronic kidney disease [220], Crohn disease and ulcerative colitis [221]. Oral health, including periodontal status, also influences these measurements. Periodontitis has been linked to increased salivary IL-6 [222,223], TNF-α [223,224] and CRP [225], as well as elevated oxidative stress markers and reduced antioxidant concentrations [226]. Oxidative stress in saliva may additionally be associated with oral squamous cell carcinoma, oral lichen planus and leukoplakia [227]. Therefore, both the patient’s systemic health and oral status must be considered when interpreting salivary biomarkers.

**Figure 2:**
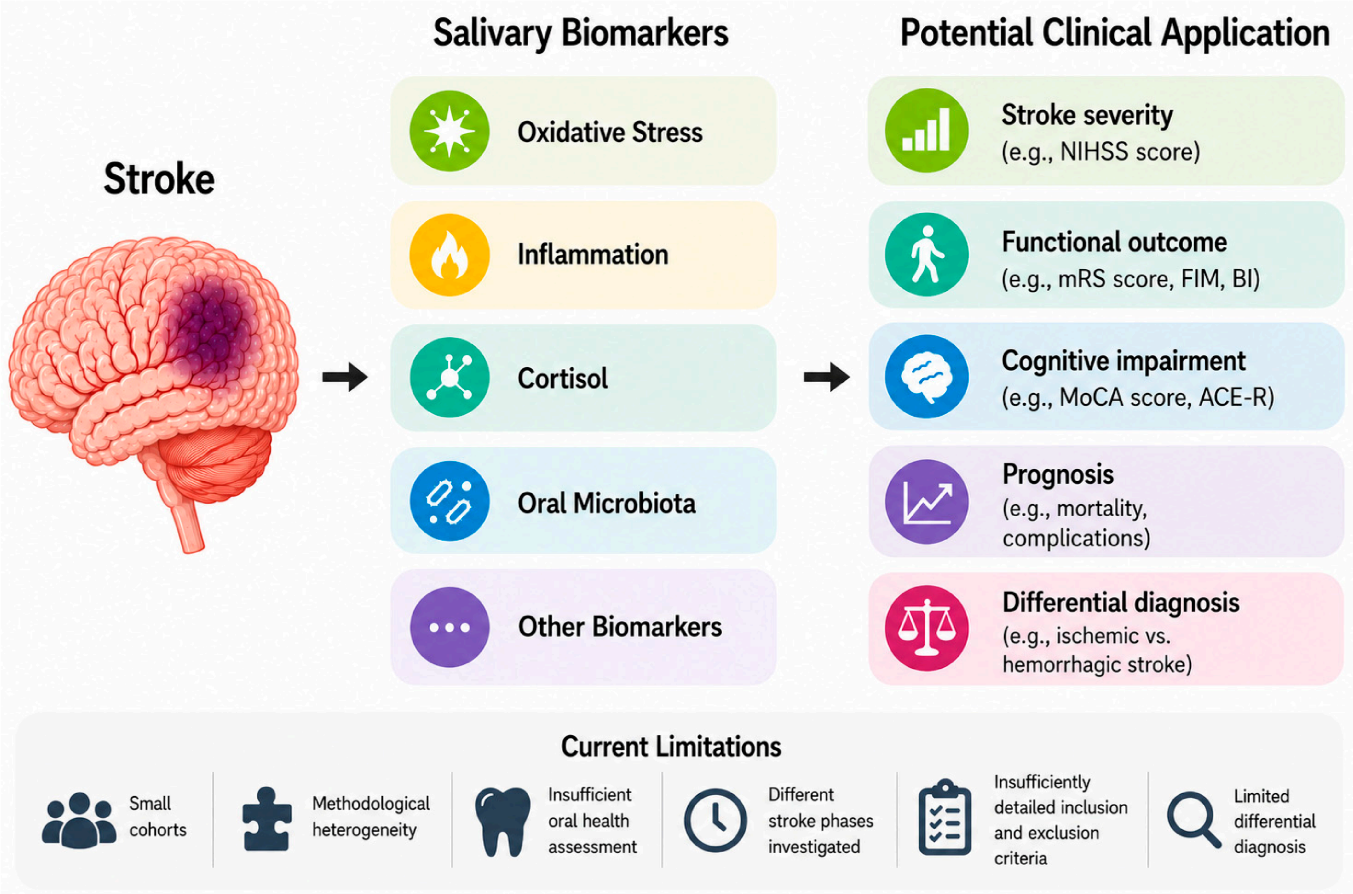
Summary of stroke biomarkers in saliva, their potential clinical applications, and the main limitations of the available evidence. ACE-R—Addenbrooke’s Cognitive Examination Revised; BI—Barthel Index; FIM—Functional Independence Measure; MoCA—Montreal Cognitive Assessment; mRS—modified Rankin Scale; NIHSS—National Institutes of Health Stroke Scale.

Most studies included in this review were observational cross-sectional studies or case-control in design. Case-control studies face challenges in selecting appropriate comparison groups and controlling for confounding variables. Verification of patient-reported data is frequently limited [228], with participants exaggerating or minimizing their risk factors [229]. Cross-sectional studies, by contrast, are limited in establishing causality or temporal relationships between risk factors and outcomes [230]. In this review, inclusion and exclusion criteria were sometimes insufficiently specified. Methods of saliva collection, including type, timing, centrifugation, and storage, varied across studies. Analytical methods for the same biomarkers differed, preventing direct comparison of results. Individual factors such as comorbidities, medications, smoking, as well as local factors like oral health, may further influence salivary composition. Only a few studies considered oral health during saliva collection, and comprehensive dental or periodontal examinations were generally lacking, despite the potential influence of oral conditions on saliva sample quality and biomarker measurements. Moreover, most studies focused exclusively on a single stroke subtype, predominantly ischemic stroke, whereas hemorrhagic stroke and direct comparisons between different stroke subtypes were rarely investigated, limiting the universality of the available evidence. The available studies compared stroke patients with healthy controls or evaluated associations between salivary biomarkers and stroke severity, functional outcomes, or post-stroke complications. Therefore, the current evidence is insufficient to determine whether these biomarkers can be used for the differential diagnosis of stroke in clinical practice. Only a few studies compared different cerebrovascular conditions, such as ischemic stroke and transient ischemic attack, while comparisons with hemorrhagic stroke, stroke mimics, or other neurological and inflammatory diseases are still lacking. Future studies should focus on these clinically relevant comparisons to better establish the diagnostic value of salivary biomarkers and should be designed as randomized clinical trials with larger cohorts of stroke survivors. Currently, the available studies suggest the potential value of selected salivary biomarkers for several clinical applications, however, methodological limitations and the heterogeneity of the existing evidence make it difficult to draw definitive conclusions regarding their clinical utility. It should be emphasized that reference values for most salivary biomarkers are also lacking, limiting their diagnostic application. Analytical methods have not been fully validated for saliva, and uniform guidelines for collection are absent, contributing to variability across studies. Substantial heterogeneity among the included studies precluded the use of advanced statistical techniques for data analysis, thereby limiting the reliability and quality of the conclusions. A summary of the reviewed studies, including the investigated salivary biomarkers, their potential clinical applications, and the main limitations of the available evidence, is presented in Figure 2.

## 8. Conclusions

This review underscores the potential clinical applications of salivary biomarkers in stroke. Saliva collection is minimally invasive, reduces patient anxiety, and is more cost-effective than blood sampling. Its accessibility and ease of collection further enhance its utility. Salivary cortisol appears to be a promising marker for prognostic assessment, whereas markers of oxidative stress and inflammation may have particular value as a tool for evaluating functional outcome, cognitive impairment, and potentially differential diagnosis. Nevertheless, these biomarkers largely represent nonspecific biological responses that are not unique to stroke and may be influenced by other neurological and systemic diseases. The available research on the use of saliva in stroke patients is scarce and limited. Many studies have focused on only one stroke subtype, without comparison to stroke-mimicking conditions or consideration of how oral health and smoking may affect salivary biomarkers. Additionally, standardized collection protocols and reference values for most salivary biomarkers remain unavailable. Future research should focus on larger populations, validation of saliva-specific analytical methods, and comprehensive assessment of clinical utility. Saliva is an increasingly recognized diagnostic medium; however, further research is needed to fully define its potential.

## Data Availability

No new data were created or analyzed in this study.

## Supplementary Materials

The following supporting information can be downloaded at: https://www.mdpi.com/article/10.3390/ijms27156871/s1.

## Abbreviations

The following abbreviations are used in this manuscript:

ACE	angiotensin-converting enzyme;
ACE-R	Addenbrooke’s Cognitive Examination Revised;
ADL	Activities of Daily Living;
AGE	Advanced Glycation End products;
AIS	Acute Ischemic Stroke;
AOPP	Advanced Oxidation Protein Products;
BDI	Beck Depression Inventory II;
BDNF	Brain-Derived Neurotrophic Factor;
BI	Barthel Index;
BP	Blood Pressure;
C	Stroke Mimics Patients;
CAT	Catalase;
CCL2/MCP-1	Chemokine (C-C motif) Ligand 2/Monocyte Chemoattractant Protein-1;
CCL3/MIP-1α	Chemokine (C-C motif) Ligand 3/Macrophage Inflammatory Protein-1 alpha;
CCL4/MIP-1β	Chemokine (C-C motif) Ligand 4/Macrophage Inflammatory Protein-1 beta;
CCL5/RANTES	Chemokine (C-C motif) Ligand 5/Regulated on Activation, Normal T-cell Expressed and Secreted;
CCL7/MCP-3	Chemokine (C-C motif) Ligand 7/Monocyte Chemoattractant Protein-3;
CCL11/eotaxin	Chemokine (C-C motif) Ligand 11/Eotaxin;
CCL27/CTACK	Chemokine (C-C motif) Ligand 27/Cutaneous T-cell-Attracting Chemokine;
CDR	Clinical Dementia Rating;
CES	Cardioembolic Stroke;
CIS	Cerebral Ischemic Stroke;
CIS-MCI	Cerebral Ischemic Stroke with Mild Cognitive Impairment;
CRP	C-Reactive Protein;
CT	Computed Tomography;
CXCL1/GRO-α	Chemokine (C-X-C motif) Ligand 1/Growth-Regulated Alpha Protein;
CXCL8/IL-8	Chemokine (C-X-C motif) Ligand 8/Interleukin-8;
CXCL9/MIG	Chemokine (C-X-C motif) Ligand 9/Monokine Induced by Gamma Interferon;
CXCL10/IP-10	Chemokine (C-X-C motif) Ligand 10/Interferon Gamma-Induced Protein 10;
CXCL12/SDF-1α	Chemokine (C-X-C motif) Ligand 12/Stromal Cell-Derived Factor-1 alpha;
DBP	Diastolic Blood Pressure;
DLMO	Dim Light Melatonin Onset;
EIA	Enzyme Immunoassay;
FGF	Fibroblast Growth Factor (basic);
FIM	Functional Independence Measure;
Fluo-HPLC	High-Performance Liquid Chromatography with Fluorescence Detection;
GCF	Gingival Crevicular Fluid;
G-CSF	Granulocyte Colony-Stimulating Factor;
GM-CSF	Granulocyte-Macrophage Colony-Stimulating Factor;
GSH	Glutathione;
H_2_O_2_	Hydrogen Peroxide;
HDRS	Hamilton Depression Rating Scale;
HGF	Hepatocyte Growth Factor;
HR	Heart Rate;
HS	Hyposalivation;
ICP-MS	Inductively Coupled Plasma Mass Spectrometry;
ICH	Intracerebral Hemorrhage;
IFN-α2	Interferon Alpha-2;
IFN-γ	Interferon Gamma;
IL-1α	Interleukin-1 alpha;
IL-1β	Interleukin-1 beta;
IL-1ra	Interleukin-1 Receptor Antagonist;
IL-2	Interleukin-2;
IL-2Rα/CD25	Interleukin-2 Receptor Alpha/Cluster of Differentiation 25;
IL-3/MCGF	Interleukin-3/Mast Cell Growth Factor;
IL-4	Interleukin-4;
IL-5	Interleukin-5;
IL-6	Interleukin-6;
IL-7	Interleukin-7;
IL-9	Interleukin-9;
IL-10	Interleukin-10;
IL-12 p40	Interleukin-12 p40 Subunit;
IL-12 p70	Interleukin-12 p70 Heterodimer;
IL-13	Interleukin-13;
IL-15	Interleukin-15;
IL-16	Interleukin-16;
IL-17	Interleukin-17;
IL-18	Interleukin-18;
IQCODE	Informant Questionnaire on Cognitive Decline in the Elderly;
LDL-C	Low-Density Lipoprotein Cholesterol;
LDL	Low-Density Lipoprotein
LH	Left Hemisphere;
LIF	Leukemia Inhibitory Factor;
LOOH	Lipid Hydroperoxides;
MALDI-TOF MS	Matrix-Assisted Laser Desorption Ionization–Time of Flight Mass Spectrometry;
MCI	Mild Cognitive Impairment;
M-CSF	Macrophage Colony-Stimulating Factor;
MDA	Malondialdehyde;
MDT	Mirror Drawing Test;
MEPs	Motor-Evoked Potentials;
MIF	Macrophage Migration Inhibitory Factor;
MMSE	Mini-Mental State Examination;
MMP-8	Matrix Metalloproteinase-8;
MoCA	Montreal Cognitive Assessment;
MPO	Myeloperoxidase;
mRS	modified Rankin Scale;
N.d.	No data;
NBT	Nitro Blue Tetrazolium;
NGF-β	Nerve Growth Factor beta;
NIHSS	National Institutes of Health Stroke Scale;
NO	Nitric Oxide;
NS	Normal Salivary Secretion;
NSE	Neuron-Specific Enolase;
NVAF	Nonvalvular Atrial Fibrillation;
NYHA II	New York Heart Association II;
NWS	Non-Stimulated Whole Saliva;
OSI	Oxidative Stress Index;
OTUs	Operational Taxonomic Units;
P	Stroke Patients with Pneumonia;
PB	Practical Baseline;
PC	Protein Carbonyls;
PDGF-BB	Platelet-Derived Growth Factor, Isoform BB;
PES	Pharyngeal Electrical Stimulation;
PFO	Patent Foramen Ovale;
PI	Plaque Index;
PSD	post-stroke dementia;
PSOD	post-stroke patients with dysphagia;
PSnOD	post stroke patients without dysphagia;
Px	Peroxidase;
RANKL	Receptor Activator of Nuclear Factor κB Ligand;
RH	Right Hemisphere;
RIA	Ruptured Intracranial Aneurysm;
SARS-CoV-2	Severe acute respiratory syndrome coronavirus 2;
SCF	Stem Cell Factor;
SCGF-β	Stem Cell Growth Factor beta;
SBS	Sitting Balance Scale;
SBP	Systolic Blood Pressure;
SM	Streptococcus mutans;
SOD	Superoxide Dismutase;
SP	Substance P;
SWS	Stimulated Whole Saliva;
TAC	Total Antioxidant Capacity;
TAG	Triacylglycerols;
TC	Total Cholesterol;
TIMP-1	Tissue Inhibitor of Metalloproteinases-1;
TNF-α	Tumor Necrosis Factor alpha;
TNF-β	Tumor Necrosis Factor beta;
TOS	Total Oxidant Status;
TPC	Total Protein Content;
TRAIL	Tumor Necrosis Factor-Related Apoptosis-Inducing Ligand;
TIA	Transient Ischemic Attack;
TSST	Trier Social Stress Test;
UA	Uric Acid;
UIA	unruptured intracranial aneurysm;
VEGF	Vascular Endothelial Growth Factor;
WAB-AQ	Western Aphasia Battery Aphasia Quotient;
WHO	World Health Organization;
XO	Xanthine Oxidase
