# Supplementary material for: Potential of Saliva in Stroke Patients: A Review
Source: Int J Mol Sci. 2026 Jul 31;27(15):6871. doi: 10.3390/ijms27156871 (PMC13466623; doi:10.3390/ijms27156871)
Supplement: Supplementary file 1 [file ijms-27-06871-s001.zip › Table S1 new 22.7.pdf]

Table S1. General characteristics of the included articles.

Author, Year	Setting	Study Group F/M Age	Control Group F/M Age	Inclusion Criteria	Exclusion Criteria	First or recurrent stroke	Type	Smoking Status F/M	Pharmacological treatment	Time of collection	Biomarkers
1.Ahmed et al. 2004 [140]	Sweden	58<br>19/39<br>39–86 yrs<br>mean: 66	571 reference population	-ischemic stroke within the time limit of 24 h after symptom onset	-radiologically confirmed primary intracerebral or subarachnoid hemorrhage<br>-previous stroke with significant residual functional deficit mRS ≥2 prior to present stroke<br>-inclusion in any other interventional study<br>-sustained coma<br>-very severe stroke with reduced consciousness<br>-other severe life-threatening concurrent illness	N.d.	Ischemic	32 patients with history of smoking	-anti-hypertensive drugs<br>-beta-blockers<br>-ACE inhibitor<br>-diuretics<br>-calcium channel blockers	at inclusion,<br>on the evening of the inclusion day 8:00pm-10:00pm,<br>on the morning of the next day 7:00am–9:00am,<br>on the afternoon of the inclusion day/<br>next day 3:00pm–5:00pm within 24 h after inclusion in the study	Cortisol
2.Laures-Gore et al. 2007	USA	15<br>3/12<br>41-71	15<br>3/12<br>41-71 yrs	-radiologically diagnosed with stroke	-radiologically negative diagnosed with stroke	First	not specified	N.d.	drug associated with estrogen, drugs for diabetes	1:30pm- 6:00pm<br>2 experimental sessions: Trier	Cortisol

[141]		yrs<br>mean:<br>54.4±9<br>.17	mean:<br>53.6±9.4	-aphasia	-no aphasia<br>-cortisol values were identified as outliers<br>-corticosteroid medications uptake					Social Stress Test (TSST) or Mirror Drawing Test (MDT)<br>within 2 weeks period<br>collection<br>after 30min of relax<br>10min after task explanation<br>10 min after performing task, every 10min for 90min	
3.Al-Rawi et al., 2008<br>[95]	Iraq	50<br>26/24<br>45-75 yrs<br>mean:<br>58.2	75 risk group:<br>25 patients with hypertensi on<br>13/12<br>25 patients with diabetes type 2<br>14/11<br>25 patients with ischemic	-clinically and radiologically diagnosed with ischemic stroke	-other systemic or local disease that may affect parameters<br>-active oral inflammation<br>-advanced periodontitis<br>-severe gingivitis	N.d.	Ischemic	26 smokers	N.d.	8:00am-9:00am	GSH<br>MDA<br>SOD<br>UA

			heart disease 14/11<br>25 healthy controls 13/12								
4.Al-Rawi, Atiyah, 2009 [128]	Iraq	50<br>26/24<br>45-75 yrs<br>mean: 58.2	75 risk group:<br>25 patients with hypertension 13/12<br>25 patients with diabetes type 2 14/11<br>25 patients with ischemic heart disease 14/11<br>25 healthy controls 13/12	-clinically and radiologically diagnosed with ischemic stroke	-other systemic or local disease that may affect parameters<br>-active oral inflammation<br>-advanced periodontitis<br>-severe gingivitis	N.d.	Ischemic	26 smokers 6/20	N.d.	8:00am-9:00am	NSE
5.Lueken et al., 2009 [150]	Germany	32<br>15/17<br>mean:	30<br>12/18	-a first-ever stroke<br>-ischemic,	-a previous history of transient ischaemic attacks	first	Ischemic	not included into study	N.d	basal:<br>6:00am-8:00am	Cortisol

		56.6 ± 8.4<br>18 LH<br>14 RH	mean: 57.8 ± 9.3	supratentorial and unilateral stroke<br>-ability to participate in the study protocol	-bilateral, brainstem or cerebellar lesions<br>-other neurological disorders -prior or present psychiatric disorders					during 3 consecutive days not including weekends<br>Phasic response: 3 pm<br>task time 10 minute<br>T1 - first baseline collection<br>T2 - second baseline collection, 10 minutes after T1, practical baseline (PB)<br>T3-T9 immediately after task completion and within 10 minutes intervals for 60 minutes	
6.Al-Rawi, 2010<br>[96]	Iraq	50<br>26/24<br>mean: 58.2	25<br>13/12	-ischemic stroke survivors	-other systemic or local disease that may affect parameters<br>-active oral inflammation<br>-advanced periodontitis	N.d.	Ischemic	26 smokers<br>6/20	N.d.	8:00am-9:00am	MDA

					-severe gingivitis						
7.Haba et al., 2011 [119]	Roma nia	103<br>20-50 yrs divide d into:<br>B- 50 patien ts with period ontal diseas e<br>23/27<br>C- 53 patien ts with period ontal diseas e and transi ent ische mic attack TIA<br>26/27	A- 40<br>20/20<br>20-50 yrs	N.d.	-risk factors for CRP changes	N.d.	TIA	N.d.	N.d.	N.d.	CRP<br>IL-6

8.Laures-Gore, 2012 [142]	USA	31 subgroups: 19 LH 11/8 mean: 55.7 ± 9.1 12 RH 4/8 mean: 58.5 ± 8.4	N.d.	-no history of multiple strokes<br>-no head injury per caretaker or self-report<br>-oral nutrition receivers<br>-no corticosteroid drugs uptake<br>-no radiologically diagnosed bilateral strokes<br>-ability to complete the entire study<br>-no development of visual impairment<br>-radiological report available	-history of multiple strokes<br>-head injury per caretaker or self-reported<br>-non-oral nutrition receivers<br>-corticosteroid drugs uptake<br>-radiologically diagnosed bilateral strokes<br>-inability to complete the entire study due to recurrent stroke<br>-development of visual impairment<br>-no radiological report available<br>-withdrawal due to the study's time commitment or other personal issues	first	not specified	N.d.	N.d.	6 samples collected biweekly for 3 months, in the afternoon	Cortisol
9.Palm et al., 2014 [127]	Germany	98, 45/53 18-80 yrs	100 47/53 18-80 yrs mean: 69.1 ± 5.2	-first-ever ischemic stroke	-clinical or laboratory signs of acute infection at time of stroke onset<br>-recurrent stroke	first	ischemic	28 current smokers 58 former smokers	-antihypertensive drugs<br>-anti-diabetics drugs<br>-cholesterol lowering drugs	within 24 hours from admission	IL-1β MMP-8 MPO TIMP-1

		mean: 68.2 ± 9.7									
10. Kwon et al., 2015 [149]	Republic of Korea	28 stroke patients with the poststroke depression 13/15 mean: 62.5 ± 7.9	23 caregivers 17/6 mean: 58.2 ± 6.7	-stroke diagnosed with MRI<br>-stroke occurrence at least 2 months prior the study<br>-scores higher than 14 on BDI or 7 on the HDRS<br>-adherence to the sampling methods<br>-sufficient amount of saliva	-steroid or antidepressants drugs intake<br>-nonadherence to saliva collection method<br>-insufficient amount of saliva	N.d.	not specified	N.d.	N.d.	immediately after awakening at 6am<br>15 min<br>30 min<br>45 min after awakening for 2 following days	Cortisol
11.Muhle et al., 2017 [172]	Germany	23 11/12 mean: 64.43± 11.7	N.d.	-patients completely weaned from ventilator<br>-able to stay alert >15 min<br>-tracheotomized patients<br>-severe and persistent dysphagia that	-decannulated before study inclusion<br>-discharged before study inclusion<br>-unable to stay alert >15 min<br>-declined to participate<br>-implanted electronic device	first+recurrence	not specified	N.d.	-i.v. thrombolysis<br>-thrombectomy<br>-external ventricular drainage<br>-decompressive craniectomy/hematoma evacuation<br>-i.v. antibiotics<br>-ACE inhibitor treatment	before and right after treatment session<br>first treatment cycle: 20 patients<br>second treatment cycle: 18<br>third treatment cycle: 15 patients<br><br>(Treatment cycle	SP

				would not allow for decannulation	-other reasons					3 days with sessions; evaluation the following day; in case of failure next treatment in 48-72h)	
12.Tong et al. 2017 [180]	China	346 mean: 75.55 ± 2.34 subgroups: 61 stroke 65 PSD 220 Alzheimer Disease mean: 75.55 ± 2.34	231	-stroke within the last 3 months to 2 years	-refusal to provide samples of blood and urine<br>-life-threatening illness<br>-inability to participate in the assessment	N.d.	not specified	N.d.	N.d.	In the morning	Formaldehyde
13.Inenaga et al., 2018 [158]	Japan	429 202/227	79 46/33 mean: 63.9 ± 9.4	N.d.	N.d.	N.d.	ischemic, hemorrhagic and other	S.mutans (-), Cnm-negative 43/185 S.mutans	-antihypertensive drugs<br>-hypoglycemic drugs<br>-insulin	immediately after admission and before the antibiotics intake	Microbiological analysis

		mean: 68.1±12.7 subgroups: 48 patients with CES 19/29 mean: 75.8±10.0 including 34 patients with NVAF 14/20 mean: 77.0±10.1 151 patients with non-CES infarcts						(+), Cnm-negative 32/171 S.mutans (+), Cnm-positive 19/73	-antibiotics		

		65/86 mean: 72.3±10.3<br>54 patients with ICH<br>21/33 mean: 69.2±11.8<br>43 patients with RIA<br>22/21 mean: 59.7±14.6<br>97 patients with UIA<br>64/33 mean: 63.1±11.4									

		37 the others , 11/26 mean: 62.6±14.7									
14.Niimi et al. 2018 [173]	Japan	40 divided into: Low SP 23 8/15 mean: 65.5 ± 10.9 High SP 17 6/11 60.2 ± 14.0	N.d.	-age <80 years	-age > 80 years<br>-taking sedative drugs<br>-taking ACE inhibitors	first+recurrence	not specified	N.d.	N.d.	N.d.	SP
15.Tene et al. 2018 [144]	Israel	182 82/100 mean: 66.8±9.6 yrs<br><br>divided into tertile	N.d.	-age >50 years -first-ever AIS or TIA<br>-admission within 72h after stroke onset<br>-a total score on the NIHSS<17	-hemorrhagic stroke<br>-cognitive impairments before the stroke (established by the short version of the IQCODE with the score>3.3)	first	ischemic	40 smokers	N.d.	bedtime and 30 minutes after awakening in the morning after admission, 7:30 am<br>6, 12, 24 months after the stroke	Cortisol

		s according to bedtime cortisol levels			-severe disability or aphasia which preclude the evaluation and follow-up<br>-drug abuse<br>-alcohol abuse<br>-endocrine/psychiatric disease<br>-steroid drugs intake within last 6 months						
16Malik et al., 2019 [58]	Malaysia	53<br>20/33<br>20+yrs	Not included into study	-stroke survivors under the management of rehabilitation physicians team<br>-functional dependency level >70 -<br>medically stable<br>-not edentulous, minimum of 20 teeth present	-patients on antibiotics or antimicrobial agents	N.d.	ischemic, hemorrhagic	15 smokers	-antiplatelet drugs e.g. aspirin<br>-anti-cholesterol drugs e.g. simvastatin<br>-anti-hypertensive drugs e.g. amlodipine<br>-beta blocker	N.d.	CRP<br>IL-6
17Mirzaee et al., 2019 [143]	Iran	12<br>0/12<br>mean: 56.5±6	12<br>0/12<br>mean: 51.6±3	-age between 18 and 70 years<br>-ischemic stroke > 1 year before the study	-age <18 and >70 years<br>-ischemic stroke < 1 year before study<br>-no radiological	N.d.	ischemic	N.d.	N.d.	10:00am-3:00pm x4 times:<br>-Before TSST,<br>-Right after TSST<br>-After two	Cortisol

				-radiological diagnosis of stroke<br>-mRS <=3<br>-no other systemic diseases<br>-no regular neurophysiologic medication uptake<br>-no smoking at least one day before the study -no heavy exercise at least one month before the study	diagnosis of stroke<br>-mRS >3<br>-other systemic diseases<br>-regular neurophysiologic medication uptake<br>-smoking less than one day before the study<br>-heavy exercise less than one month before the study					recoveries: 20 and 40 minutes after TSST	
18Cieplik et al., 2020 [157]	Germany	57 subgroups: 49 stroke patients 15/34 59-78 yrs median:71 8 stroke	42 stroke mimics patients (C) 24/18 51.3-78.3yrs median:66.5	-clinically or radiologically diagnosed stroke lasting >24h	-intubation at time of admission<br>-endocarditis prophylaxis<br>-recurrence of stroke within 1 month	first+recurrence	ischemic	active smokers:<br>S:24<br>P:4<br>C:16	-embolectomy<br>-nasogastric tube<br>-antibiotics	-within 24 h of admission (baseline)<br>-48h after baseline investigation<br>-120h after baseline investigation	Microbiological analysis

		patients with pneumonia (P) 3/5 71.8-85.8yrs median:79.5									
19Gerreth et al., 2020 [13]	Poland	30 12/18 34-84 yrs mean: 61.87 ± 11.81	30 12/18 mean: 61.87 ± 11.81	-age >18 years -good general condition; -radiologically confirmed cerebral infarction or cerebral hemorrhage -recovery from acute phase of ischemic or hemorrhagic stroke in all brain areas -first admission to cure stroke unit more than 5–6 (to 10) h from	-age <18 years -radiologically unconfirmed cerebral infarction or cerebral hemorrhage -ischemic stroke treated with thrombolysis or thrombectomy -stroke recurrence during subacute phase -unconsciousness and inability to give informed consent for oral check-up and saliva sampling	N.d.	ischemic, hemorrhagic	not included into study	N.d.	7:30am-9:00am	AGE AOPP CAT Concentration of LOOH OSI Px GSH SOD TAC TOS UA

				the onset of the first neurological symptoms<br>-consciousness and giving of written and informed consent for oral examination and sampling of saliva,<br>-adequate capacity to follow instructions	-legal guardianship<br>-incapability to collect saliva sample<br>-insufficient cooperation due to language/cognitive deficits<br>-heart failure resting oxygen saturation ≤ 92%<br>-autoimmune disease - cardiovascular disease<br>-lung disease<br>-women suffering from malnutrition (body mass index <18 kg/m2) or weight loss >10% during the previous 3 months<br>-psychiatric or cognitive disorders<br>-smokers<br>-taking vitamins and dietary supplements for the last 3 months						

20García-Cabo et al., 2020 [99]	Spain	20 12/8 between 61-83 yrs mean: 72 subgroups: 10 ischemic stroke 10 hemorrhagic stroke	N.d.	-patients within 48h from stroke onset -radiologically confirmed stroke	-vitamin or mineral supplement intake -nasal hemorrhage -liver disorders -working in heavy industry	N.d.	ischemic, hemorrhagic	N.d.	N.d.	within 48h from stroke onset	Iron
21 Maciejczyk et al. 2020 [61]	Poland	30 subgroups: 16 NS 5/11 34-67 yrs mean: 63.44 ± 7.74 14 HS 7/7 52-84 yrs	30 11/19 mean: 62.47 ± 10.43	-radiologically confirmed cerebral infarction or cerebral hemorrhage, -Recovery from the acute phase of ischemic or hemorrhagic stroke in all brain areas -good general condition	-radiologically unconfirmed cerebral infarction or cerebral hemorrhage -stroke recurrence during subacute phase -poor general condition, -unconsciousness and inability to give informed consent for saliva	N.d.	ischemic, hemorrhagic	not included into study	N.d.	7:30am-9:00am	Amadori products – protein glycation Amylase activity Dityrosine Kynurenine NO Nitrotyrosine concentration

		mean:<br>61.36<br>±<br>13.08		-consciousness and giving of written and informed consent for saliva sampling and oral examination<br>-age >18 years<br>-first admission to cure stroke unit more than 5–6 (to 10) hours from the onset of the early neurological symptoms,<br>-adequate capacity to follow instructions<br>-ability to collect a saliva sample	sampling and oral check-up<br>-insufficient cooperation due to cognitive/language deficits<br>-incapability to collect saliva sample<br>-age <18 years<br>-legal guardianship<br>-ischemic stroke treated with thrombectomy or thrombolysis<br>-psychiatric or cognitive disorders<br>-lung disease<br>-cardiovascular disease<br>-autoimmune disease<br>-heart failure resting oxygen saturation ≤ 92%, -women with malnutrition, i.e., having body mass index lower than 18 kg/m2 or with weight loss over 10% during the						N-formylkynurenine<br>Peroxynitrite<br>PC<br>Total thiols<br>Tryptophan

					previous 3 months<br>-smokers<br>-taking vitamins and dietary supplements for the last 3 months						
22Maciejczyk et al. 2021 [12]	Poland	25<br>13/12<br>mean: 63.52 ± 8.545	25<br>13/12<br>mean: 63.52 ± 8.545	-radiologically confirmed cerebral infarction or cerebral hemorrhage<br>-recovery from the acute phase of ischemic or hemorrhagic stroke in all brain areas<br>-consciousness<br>-giving of written and informed consent for saliva sampling and oral examination<br>-age >18 years,<br>-first admission to cure stroke unit more than 5–6 (to 10) hours from the onset of the early	-radiologically unconfirmed cerebral infarction or cerebral hemorrhage<br>-stroke recurrence during subacute phase<br>-poor general condition<br>-unconsciousness and inability to give informed consent for saliva sampling and oral check-up<br>-insufficient cooperation due to cognitive/language deficits<br>-incapability to collect saliva sample<br>-age <18 years<br>-legal guardianship	N.d.	ischemic, hemorrhagic	not included into study	N.d.	7:30am-9:00am	IL-6<br>IL-10<br>TPC<br>TNF-α

				neurological symptoms	-ischemic stroke treated with thrombectomy or thrombolysis<br>-psychiatric or cognitive disorders<br>-lung disease<br>-cardiovascular disease -autoimmune disease<br>-heart failure above NYHA II<br>-cognitive impairment before stroke<br>-patients with malnutrition, i.e., having body mass index lower than 18 kg/m2 or with weight loss over 10% during the previous 3 months<br>-smokers<br>-taking vitamins and dietary supplements for the last 3 months						
23Wang et al. 2021	Chin a	200 subgr	N.d.	-age 40-80 years<br>-the diagnosis of	-age <40 and >80 years	first	ischemic	smoking history in	N.d.	8am the next morning after	Cortisol

[146]		oups:<br>120 patients after CIS-MCI group<br>55/65<br>mean: 62.25 ± 8.33yrs<br>80 patients after cerebral ischemic stroke without cognitive impair-ment CIS group		CIS met the diagnostic criteria established in 2012<br>by quality control industry standard<br>-first episode of stroke<br>-the course of disease was 3 months<br>-patients and their family members were all informed<br>-the diagnosis of cognitive impairment after CIS met the MCI diagnostic criteria	-depression, Alzheimer's disease, and Parkinson's disease<br>-severe aphasia, blindness, and deafness,<br>-other organic diseases of central nervous system<br>-abnormal blood thyroxine levels<br>-mRS score >4 points<br>-severe mental illness			58 patients of CIS-MCI group<br>40 patients of CIS group		enrollment	

		CIS-MCI group divided according to cortisol level:<br>-the first quartile(0.85–1.55 nmol/L)<br>-the second quartile interval (1.55–2.25 nmol/L),<br>-the third									

		quartile interval (2.25–2.95 nmol/L), -the fourth quartile interval (2.95–3.65 nmol/L)									
24Dresang et al., 2022 [167]	USA	17 subgroups: 8 BDNF Val66Val carriers mean: 54.8 ±13.9 2/6	N.d.	-stroke occurred >6 months prior to study	-stroke occurred <6 months prior to study<br>-scoring higher than the WAB-AQ cutoff for aphasia diagnosis (1 person excluded)<br>-Met66Met homozygote	N.d.	ischemic	N.d.	N.d.	N.d.	BDNF

		10 Val66 Met allele carriers 0/10 58 ±13.9									
25 Maciejczyk et al. 2022 [69]	Poland	30 subgroups: 15 ischemic stroke 8/7 mean: 61.6 ± 12.97 15 hemorrhagic stroke 8/7 mean: 64.53 ± 8.123	30 15/15 mean: 63.07 ± 10.74	-radiologically confirmed cerebral infarction or cerebral hemorrhage -good general condition, -age >18 years -no legal guardianship -recovery from the acute phase of ischemic or hemorrhagic stroke in all brain areas -first admission to cure stroke unit more than 5–6 (to 10) hours	-radiologically not confirmed cerebral infarction or cerebral hemorrhage -poor general condition -the age <18 years - legal guardianship -no recovery from the acute phase of ischemic or hemorrhagic stroke in all brain areas -first admission to cure stroke unit less than 5–6 hours from the onset of the early neurological	N.d.	ischemic, hemorrhagic	not included into study	N.d.	7:30am-9:00am	H_2O_2 TPC UA XO activity

				from the onset of the early neurological symptoms<br>-consciousness and giving of written and informed consent for saliva sampling and oral examination<br>-adequate capacity to follow instructions<br>-ability to collect a saliva sample<br>-no stroke recurrence during the subacute phase<br>-ischemic stroke treated without thrombectomy or thrombolysis<br>-patients without malnutrition, i.e., having body mass index lower than 18 kg/m2 or with weight loss over 10% during the previous 3	symptoms and treated with thrombolysis<br>-unconsciousness and unable to give written and informed consent for saliva sampling and oral examination<br>-inadequate capacity to follow instructions<br>-inability to collect a saliva sample<br>-stroke recurrence during the subacute phase<br>-ischemic stroke treated with thrombectomy or thrombolysis<br>-patients with malnutrition, i.e., having body mass index lower than 18 kg/m2 or with weight loss over 10% during the previous 3 months<br>-heart failure<br>-autoimmune						

				months<br>-no heart failure<br>-no autoimmune disease<br>-no psychiatric or cognitive disorders<br>-no lung disease<br>-no XO inhibitors<br>-no vitamins and dietary supplements taken for the last 3 months<br>-nonsmokers	disease<br>-psychiatric or cognitive disorders<br>-lung disease<br>-XO inhibitors for the last 3 months<br>-vitamins and dietary supplements taken for the last 3 months<br>-smokers						
26 Pawlukowska et al. 2022 [126]	Poland	100<br>40/60<br>46-75 yrs<br>mean: 66.1 ± 9.22<br><br>Group I: with stimulation of salivary gland	No data	-first ischemic stroke with symptoms from the anterior cerebral artery<br>-a significant neurological deficit minimum 3 points according to NIHSS	-aphasia<br>-disturbances of consciousness<br>-mental disorders impossible to express informed consent<br>-surgery of the salivary glands<br>-disrupting the secretion of saliva<br>-diseases that disrupt salivary secretion	first	ischemic	N.d.	N.d.	1st and 7th day of stroke	IL-1β<br>MMP-8<br>OPG<br>RANKL

		s and oral hygiene - 54 Group II: no treatment - 46									
27 Zhanina et al., 2022 [147]	Russia	45 12/33 mean: 56 ± 5<br><br>divided into: - subgroups with and without cognitive impairment - subgroups with	32 13/19 mean: 57 ± 4 yrs	-age 45–80 years; -hemispheric cerebral ischemia, not involving limbic structures -mild to moderate stroke severity (1–15 points on the US NIHSS) - admission to the hospital within 48 h after stroke onset	-previous stroke -craniocerebral injury with residual focal changes on CT/MRI or accompanied by loss of consciousness in the history -the history of previous cognitive and depressive disorders -presence of acute or chronic somatic and hormonal diseases -alcohol or drug addiction	first	ischemic	N.d.	-drugs lowering blood pressure -drugs which cause the dissolution of the vascular thrombus -antioxidants - nootropics - vasodilators - antiarrhythmic drugs	-in the acute period after stroke (45 patients) -30 days after stroke (41 patients) -180 days after stroke (33 patients) -365 days after stroke (29 patients)<br><br>saliva collection between 14:30 and 15:30	α-amylase cortisol

		and without depressive disorder									
28 Alvarez-Larruy et al., 2023 [174]	Spain	45 17/28 mean: 71.78± 13.46 subgroups: 18 PSnOD 5/13 mean: 64.22± 11.37 27 PSOD 12/15 mean: 76.81± 12.34	N.d.	-age >=18 years -clinically and radiologically diagnosed hemorrhagic or ischemic unilateral stroke -no more than 15 days from stroke onset -SARS-COV2 negative test	-a life expectancy of less than 3 months -severe dementia (Global Deterioration Scale > 5) -suspected or confirmed SARS-COV-2 infection	N.d.	ischemic, hemorrhagic	N.d.	N.d.	in the morning 8:00am-12:00pm in the afternoon of the same day 1:30pm-5:00pm	SP
29 Manzoor et al., 2024 [159]	Finland	155 cryptogenic	153 controls 62/91	-age 18–49 years -first-ever imaging-positive	-antibiotics intake -no information on antibiotic use	first	ischemic	74 smokers in the study group	N.d.	N.d.	Microbiological Analysis

		ischemic stroke patients 65/90 median<br><br>41.81 between 34.4 and 45.8 yrs	median 42.10 between 34.5 and 46.8 yrs	acute ischemic stroke	history<br>-high microbiome reads			62 smokers in the control group			
30<br>Atam et al., 2024<br>[151]	India	75 stroke patients 36/39 mean: 62.67 ± 12.43	75<br>21/54<br>mean: 54.23 ± 13.93	-age over 12 years<br>-acute stroke meeting the WHO definition of stroke<br>-admission within six hours of the stroke episode	-hemorrhagic stroke<br>-clinical and radiological findings inconsistent with stroke<br>-head injury<br>-intracranial space-occupying lesion<br>-chronic kidney disease	N.d.	ischemic	21 smokers in the study group<br>20 smokers in the healthy controls	N.d.	between 6:00 AM and 9:00 AM	Cortisol

31Roongpiboonsopit et al., 2025 [162]	Thailand	41 AIS and TIA patients 15/26 5/13 mean: 65.31±9.46	20 9/11 5/13 mean: 62.85±7.70	-age ≥ 45 years, -onset of stroke symptoms < 72 hours -admission to the stroke unit -available CT or MRI -written informed consent	-altered consciousness or inability to provide consent, -history of stroke (controls only) - recent antibiotic use -active infection -temperature > 38°C -white blood cell count > 15,000 cells/mm3 -cancer history -abnormal kidney function (serum creatinine > 2 mg/dL) -autoimmune diseases, and -pregnancy or lactation	N.d.	ischemic, TIA	14 smokers	N.d.	the control group: in the morning before breakfast stroke group: within 72 hours of stroke onset (before any meal)	Microbiological Analysis
32Tonomura et al., 2025 [161]	Japan	189 saliva samples from 151 subjects with	55 saliva samples from 53 subjects 28/25 mean: 74.4±6.9	-age >20 years -admitted within 1 day of symptom onset -subject or relatives provided written informed consent	-antibiotics use within the past 3 months based on a systematic review -defecated after admission but before recruitment	first and recurrence.	ischemic	79 smokers in study group 7 smokers in control group	N.d.	within 3 days of admission	Microbiological Analysis

		acute ischemic stroke 61/90 mean: 72.4±12.7									
33Manzoor et al., 2025 [160]	Finland	104 subgroups: 52 cryptogenic ischemic stroke patients with high-risk PFO 13/36 mean: 41.01±6.5<br><br>52 crypto	16 stroke-free controls with high-risk PFO	-age 18–49 years<br>-first-ever imaging-positive acute ischemic stroke	-antibiotics intake within one-month preceding saliva sample collection<br>-no information on antibiotic use history	first	ischemic	21 cryptogenic ischemic stroke patients with high-risk PFO 30 smokers within cryptogenic ischemic stroke patients without PFO	N.d.	N.d.	Microbiological Analysis

		genic ische mic stroke patien ts witho ut PFO 23/29 mean:<br><br>36.98± 9.2									
34Forszt et al., 2025 [114]	Polan d	22<br>9/13<br>betwe en 53 and 80 yrs	22<br>9/13<br>between 53 and 80 yrs	-radiological confirmation of ischemic stroke based on the WHO standards<br>-the first stroke in a patient's life<br>-age over 18 years<br>-ability to give informed consent for the study and saliva sample collection<br>-the ability to provide a saliva sample	-history of second or subsequent stroke -autoimmune (e.g., diabetes, Hashimoto's disease, psoriasis, rheumatoid arthritis, systemic sclerosis) or psychiatric diseases (e.g., Alzheimer's disease, Parkinson's disease)<br>-prior acute	first	ischemic	not included into study	17 stroke patients received < 5 drugs/day<br>5 stroke patients received > 5 drugs/day	within the first 2 days after admission to the ward, between 10 and 13 days following the stroke incident	Pro-inflammatory cytokines:<br>IL-1α<br>IL-1β<br>IL-7<br>IL-16<br>IL-18<br>TNF-α<br>TNF-β<br><br>Anti-inflammatory cytokines:<br>IL-1ra<br>IL-10

					coronary events<br>-thromboembolic events (such as limb thrombosis or pulmonary embolism)<br>-smoking<br>-inability to give informed consent, or to provide a saliva sample						IL-13<br>TRAIL<br><br>Th1 cytokines:<br>IFN-α2<br>IFN-γ<br>IL-2<br>IL-2Rα / CD25<br>IL-12 p40<br>IL-12 p70<br>IL-15<br><br>Th2 cytokines:<br>IL-4<br>IL-5<br>IL-6<br>IL-9<br><br>Th17 cytokines:<br>IL-17
35 Forszt et al., 2025 [115]	Poland	22<br>9/13<br>median 65 yrs	22<br>9/13<br>median 65 yrs	-individuals aged 18 years or older<br>-radiologically confirmed ischemic stroke<br>-first-ever	-individuals under 18 years of age<br>-lack of radiological confirmation of ischemic stroke	first	ischemic	not included into study	17 stroke patients received < 5 drugs/day<br>5 stroke patients received > 5 drugs/day	between 8:00 and 10:00 in the morning	chemokines:<br>CCL2 / MCP-1<br>CCL3 / MIP-1α<br>CCL4 / MIP-

				ischemic stroke<br>-ability to provide informed consent for participation in the study and for saliva collection<br>-ability to provide a saliva sample<br>-no history of autoimmune, psychiatric, or thromboembolic diseases (e.g., limb thrombosis, pulmonary embolism, or recent acute coronary syndrome)<br>-no post-stroke infections<br>-no history of salivary gland diseases<br>-non-smokers<br>-stroke not treated with thrombectomy or thrombolysis	-second or subsequent stroke<br>-inability to provide informed consent for participation in the study and saliva sampling<br>-inability to provide a saliva sample<br>-presence of autoimmune, psychiatric, and/or thromboembolic diseases (e.g., limb thrombosis, pulmonary embolism, or recent acute coronary syndrome)<br>-post-stroke infections<br>-history of salivary gland diseases<br>-cigarette smoking<br>-stroke treated with thrombectomy or thrombolysis						1β<br>CCL5 / RANTES<br>CCL7 / MCP-3<br>CCL11 / eotaxin<br>CCL27 / CTACK<br>CXCL1 / GRO-α<br>CXCL8 / IL-8<br>CXCL9 / MIG<br>CXCL10 / IP-10<br>CXCL12 / SDF-1α<br>MIF<br><br>growth factors:<br>basic FGF<br>G-CSF<br>HGF<br>IL-3 / MCGF<br>LIF<br>M-CSF<br>PDGF-BB<br>VEGF

											GM-CSF<br>SCGF-β<br>SCF<br>NGF-β
36 Sun et al. 2026 [176]	Chin a	182 AIS patien ts 54/128 mean: 59.02 ± 10.06	73 controls 31/42 mean: 59.64 ± 8.61yrs	-stroke symptoms within 7 days of their first visit	-engaging in shift work or working across time zones in the past month<br>-a history of insomnia<br>-restless legs syndrome<br>-rapid eye movement sleep behavior disorder<br>-a history of neurodegenerative disorders, stroke<br>-cancer<br>-hematologic diseases -chronic inflammatory diseases<br>-severe hepatic or renal diseases<br>-autoimmune diseases<br>-pineal calcification<br>-caffeine intake > 200 mg/day<br>-a history of taking	first	ischemic	89 smokers in the study group<br>23 smokers in the control group	aspirin and statin within all patients	phase 1:<br>6 daily time points (0:00, 3:00, 6:00, 12:00, 18:00, 21:00) to assess melatonin levels, secretion curves and AUC<br>phase 2:<br>hourly samples from 19:00–23:00 to assess DLMO, Post-DLMO surge and AUC30	Melatonin

					psychotropic drugs, melatonin, or immunosuppressants within one month<br>-uncooperative patients with speech, visual, or hearing impairments and/or severe cognitive decline						

ACE – angiotensin-converting enzyme; AGE – Advanced Glycation End products; AIS – Acute Ischemic Stroke; AOPP – Advanced Oxidation Protein Products; BDI – Beck Depression Inventory II; BDNF – Brain-Derived Neurotrophic Factor; C – Stroke Mimics Patients; CAT – Catalase; CCL2 / MCP-1 – Chemokine (C-C motif) Ligand 2 / Monocyte Chemoattractant Protein-1; CCL3 / MIP-1α – Chemokine (C-C motif) Ligand 3 / Macrophage Inflammatory Protein-1 alpha; CCL4 / MIP-1β – Chemokine (C-C motif) Ligand 4 / Macrophage Inflammatory Protein-1 beta; CCL5 / RANTES – Chemokine (C-C motif) Ligand 5 / Regulated on Activation, Normal T-cell Expressed and Secreted; CCL7 / MCP-3 – Chemokine (C-C motif) Ligand 7 / Monocyte Chemoattractant Protein-3; CCL11 / eotaxin – Chemokine (C-C motif) Ligand 11 / Eotaxin; CCL27 / CTACK – Chemokine (C-C motif) Ligand 27 / Cutaneous T-cell-Attracting Chemokine; CES – Cardioembolic Stroke; CIS – Cerebral Ischemic Stroke; CIS-MCI – Cerebral Ischemic Stroke with Mild Cognitive Impairment; CRP – C-Reactive Protein; CXCL1 / GRO-α – Chemokine (C-X-C motif) Ligand 1 / Growth-Regulated Alpha Protein; CXCL8 / IL-8 – Chemokine (C-X-C motif) Ligand 8 / Interleukin-8; CXCL9 / MIG – Chemokine (C-X-C motif) Ligand 9 / Monokine Induced by Gamma Interferon; CXCL10 / IP-10 – Chemokine (C-X-C motif) Ligand 10 / Interferon Gamma-Induced Protein 10; CXCL12 / SDF-1α – Chemokine (C-X-C motif) Ligand 12 / Stromal Cell-Derived Factor-1 alpha; CT – Computed Tomography; DLMO – Dim Light Melatonin Onset; FGF – Fibroblast Growth Factor (basic); G-CSF – Granulocyte Colony-Stimulating Factor; GM-CSF – Granulocyte-Macrophage Colony-Stimulating Factor; GSH – Glutathione; H2O2 – Hydrogen Peroxide; HDRS – Hamilton Depression Rating Scale; HGF – Hepatocyte Growth Factor; HS – Hyposalivation; ICH – Intracerebral Hemorrhage; IFN-α2 – Interferon Alpha-2; IFN-γ – Interferon Gamma; IL-1α – Interleukin-1 alpha; IL-1β – Interleukin-1 beta; IL-1ra – Interleukin-1 Receptor Antagonist; IL-2 – Interleukin-2; IL-2Rα / CD25 – Interleukin-2 Receptor Alpha / Cluster of Differentiation 25; IL-3 / MCGF – Interleukin-3 / Mast Cell Growth Factor; IL-4 – Interleukin-4; IL-5 – Interleukin-5; IL-6 – Interleukin-6; IL-7 –

Interleukin-7; IL-9 – Interleukin-9; IL-10 – Interleukin-10; IL-12 p40 – Interleukin-12 p40 Subunit; IL-12 p70 – Interleukin-12 p70 Heterodimer; IL-13 – Interleukin-13; IL-15 – Interleukin-15; IL-16 – Interleukin-16; IL-17 – Interleukin-17; IL-18 – Interleukin-18; IQCODE – Informant Questionnaire on Cognitive Decline in the Elderly;] LH – Left Hemisphere; LIF – Leukemia Inhibitory Factor; LOOH – Lipid Hydroperoxides; MCI – Mild Cognitive Impairment; M-CSF – Macrophage Colony-Stimulating Factor; MDA – Malondialdehyde; MDT – Mirror Drawing Test; MIF – Macrophage Migration Inhibitory Factor; MMP-8 – Matrix Metalloproteinase-8; MPO – Myeloperoxidase; MRI – Magnetic Resonance Imaging; mRS – Modified Rankin Scale; N.d. - No data; NGF-β – Nerve Growth Factor beta; NIHSS – National Institutes of Health Stroke Scale; NO – Nitric Oxide; NS – Normal Salivary Secretion; NSE – Neuron-Specific Enolase; NVAF – Nonvalvular Atrial Fibrillation; NYHA II – New York Heart Association II; OSI – Oxidative Stress Index; P – Stroke Patients with Pneumonia; PC – Protein Carbonyls; PDGF-BB – Platelet-Derived Growth Factor, Isoform BB; PFO – Patent Foramen Ovale; PSD – post-stroke dementia; PSOD – post-stroke patients with dysphagia; PSnOD – post stroke patients without dysphagia; Px – Peroxidase; RANKL – Receptor Activator of Nuclear Factor κB Ligand; RH – Right Hemisphere; RIA – Ruptured Intracranial Aneurysm; SARS-CoV-2 – Severe acute respiratory syndrome coronavirus 2; SCF – Stem Cell Factor; SCGF-β – Stem Cell Growth Factor beta; SM – Streptococcus mutans; SOD – Superoxide Dismutase; TAC – Total Antioxidant Capacity; TIMP-1 – Tissue Inhibitor of Metalloproteinases-1; TNF-α – Tumor Necrosis Factor alpha; TNF-β – Tumor Necrosis Factor beta; TOS – Total Oxidant Status; TPC – Total Protein Content; TRAIL – Tumor Necrosis Factor-Related Apoptosis-Inducing Ligand; TIA – Transient Ischemic Attack; TSST – Trier Social Stress Test; UA – Uric Acid; UIA – unruptured intracranial aneurysm; VEGF – Vascular Endothelial Growth Factor; WHO – World Health Organization; XO – Xanthine Oxidase
