# Supplementary material for: Potential of Saliva in Stroke Patients: A Review
Source: Int J Mol Sci. 2026 Jul 31;27(15):6871. doi: 10.3390/ijms27156871 (PMC13466623; doi:10.3390/ijms27156871)
Supplement: Supplementary file 1 [file ijms-27-06871-s001.zip › Table S2 new 22.7.pdf]

Table S2. Detailed information on the investigated biomarkers.

Biomarker	Author, Year	Type of Saliva	Centrifugation and Storage	Analytical method	Results
AGE	Gerreth et al., 2020 [13]	NWS/SWS	3000xg, 20 min, + 4 °C -80°C	Fluorometrically	Significantly higher in the NWS/SWS of study group than in the control group
	Maciejczyk et al., 2020 [61]	NWS/SWS	3000xg, 20 min, + 4 °C -80°C	Fluorometrically	Significantly higher in the NWS/SWS of the HS subgroup than in the NS subgroup and control group.
AOPP	Gerreth et al., 2020 [13]	NWS/SWS	3000xg, 20 min, + 4 °C -80°C	Colorimetrically	Significantly higher in the NWS/SWS of study group than in the control group.
Amadori products – protein glycation	Maciejczyk et al., 2020 [61]	NWS/SWS	3000xg, 20 min, + 4 °C -80°C	NBT assay	Significantly higher in the NWS of the HS subgroup than the NS subgroup and control group.<br>Significantly higher in the SWS of the HS subgroup than the control group
Amylase activity	Maciejczyk et al., 2020 [61]	NWS/SWS	3000xg, 20 min, + 4 °C -80°C	Colorimetrically	Significantly lower in the NWS of both subgroups than the control group<br>Significantly lower in the SWS of the HS subgroup than the NS subgroup and the control group
	Zhanina et al., 2022 [147]	N.d.	2000× g for 15 min		Lower in the study group than control group in the acute period of stroke<br>Significant increase in the study group 30,180 and 365 days after stroke compared to acute period of stroke
BDNF	Dresang et al., 2022 [167]	saliva Oragene® DNA collection kits (NWS)	N.d.	TaqMan SNP Genotyping Assay, PCR	Decreased aphasia severity in Val66Val carriers more than Val66Met carriers<br>Increased age was related to increased aphasia severity more for Val66Val carriers than Val66Met carriers<br>Younger age was related to decreased aphasia severity for Val66Val carriers more than Val66Met carriers<br>Positive correlation between cortical excitability with WAB-AQ for Val66Val carriers<br>Negative correlation between cortical excitability with WAB-AQ for Val66Met carriers

					Less severe aphasia (higher WAB-AQ) was related to greater MEPs suppression in both BDNF genotypes, however the correlation was stronger for Val66Met carriers than Val66Val carriers
CAT	Gerreth et al., 2020 [13]	NWS/SWS	3000xg, 20 min, + 4 °C -80°C	Colorimetrically	Significantly higher in NWS/SWS of the study group than the control group
CCL2/MCP-1	Forszt et al., 2025 [115]	NWS	3000xg, 20 min, + 4 °C -80°C	immunofluorometric assay	No significant differences between the groups<br>Negative correlation with ACE-R
CCL3/MIP-1α	Forszt et al., 2025 [115]	NWS	3000xg, 20 min, + 4 °C -80°C	immunofluorometric assay	No significant differences between the groups<br>Negative correlation with ACE-R<br>Positive correlation with FIM
CCL4/MIP-1β	Forszt et al., 2025 [115]	NWS	3000xg, 20 min, + 4 °C -80°C	immunofluorometric assay	No significant differences between the groups<br>Negative correlation with ACE-R<br>Positive correlation with FIM
CCL5/RANTES	Forszt et al., 2025 [115]	NWS	3000xg, 20 min, + 4 °C -80°C	immunofluorometric assay	Below detection level
CCL7/MCP-3	Forszt et al., 2025 [115]	NWS	3000xg, 20 min, + 4 °C -80°C	immunofluorometric assay	Significantly lower in NWS of the study group than the control group<br>Positive correlation with FIM
CCL11/eotaxin	Forszt et al., 2025 [115]	NWS	3000xg, 20 min, + 4 °C -80°C	immunofluorometric assay	Significantly lower in NWS of the study group than the control group
CCL27/CTACK	Forszt et al., 2025 [115]	NWS	3000xg, 20 min, + 4 °C -80°C	immunofluorometric assay	Significantly higher in NWS of the study group than the control group<br>Negative correlation with ACE-R<br>Positive correlation with FIM<br>Positive correlation with SBS
CXCL1/GRO-α	Forszt et al., 2025 [115]	NWS	3000xg, 20 min, + 4 °C -80°C	immunofluorometric assay	No significant differences between the groups
CXCL8/IL-8	Forszt et al., 2025 [115]	NWS	3000xg, 20 min, + 4 °C -80°C	immunofluorometric assay	Significantly higher in NWS of the study group than the control group<br>Negative correlation with ACE-R

CXCL9/MIG	Forszt et al., 2025 [115]	NWS	3000xg, 20 min, + 4 °C -80°C	immunofluorometric assay	Significantly higher in NWS of the study group than the control group<br>Negative correlation with ACE-R<br>Positive correlation with FIM
CXCL10/IP-10	Forszt et al., 2025 [115]	NWS	3000xg, 20 min, + 4 °C -80°C	immunofluorometric assay	Significantly lower in NWS of the study group than the control group<br>Negative correlation with ACE-R<br>Positive correlation with FIM<br>Positive correlation with SBS<br>Positive correlation with BI
CXCL12/SDF-1α	Forszt et al., 2025 [115]	NWS	3000xg, 20 min, + 4 °C -80°C	immunofluorometric assay	No significant differences between the groups<br>Negative correlation with ACE-R<br>Positive correlation with FIM
Concentration of total LOOH	Gerreth et al., 2020 [13]	NWS/SWS	3000xg, 20 min, + 4 °C -80°C	Colorimetrically	Significantly higher in NWS/SWS of the study group than the control group
Cortisol	Ahmed et al., 2004 [140]	NWS	N.d.	‘Spectra’ cortisol 125I-coated tube radioimmunoassay	% of patients that completed saliva sampling:<br>100% at inclusion,<br>93% in the morning,<br>97% in the afternoon<br>90% in the evening<br>Abnormal and/or high salivary cortisol was correlated with higher 24-hr mean BP and HR<br>24-hr mean salivary cortisol was significantly correlated with 24-hr mean SBP, mean night-time SBP and mean night-time DBP<br>Salivary cortisol at inclusion and in the evening were equally significantly related to these BP variables<br>The morning cortisol was related to night-time SBP<br>The cortisol in the afternoon was not related to BP
	Laures-Gore et al., 2007 [141]	NWS	–60 °C	Radioimmunoassay	No significant difference over time within the study group between the linguistic and nonlinguistic task in the control group<br>Lower reactivity during the linguistic task than during the nonlinguistic task in the study group

					Lower reactivity during the linguistic task in the study group than in the control group<br>Higher stress posttask than pretask in both groups, however higher stress in the study group than the control group.
	Lueken et al., 2009 [150]	NWS	N.d.<br>-20°C	Salivary cortisol immunoassay	Basal:<br>Not significantly elevated in the study group than in the control group<br>Significantly increased only in the LH subgroup than in the control group<br>No significant differences between LH and RH subgroups.<br>A positive correlation with size lesion in LH subgroup.<br>Phasic:<br>Elevated cortisol values in the study group in T7 (i.e. 40 min after test) compared to PB value<br>Elevated cortisol values in the LH subgroup in T7 (i.e. 40 min after test) compared to PB value<br>Higher values in the RH subgroup than in the LH subgroup in T2, however not statistically different<br>Reduced measures in lesions located more anterior, especially in the RH subgroup.
	Laures-Gore 2012 [142]	NWS	N.d.<br>-80°C	Radioimmunoassa y	No significant differences between groups in cortisol levels<br>No significant differences between groups in cortisol changes over time<br>No significant differences in changes of cortisol levels in each group<br>No significant correlation between aphasia severity and cortisol levels<br>No significant correlation in the LH group between cortisol levels and naming skills<br>No(-) significant correlation in the RH group between cortisol levels and naming skills<br>No significant correlation in the RH group between cortisol levels and severity of cognitively-based language disorders

	Kwon et al., 2015 [149]	NWS	N.d.<br>-80°C	Radioimmunoassay	No significant increase at any saliva sampling time in the study group.<br>Significantly higher in the control group 15 and 30 minutes after awakening than in the study group<br>Significant differences between the two groups 15, 30 and 45 minutes after awakening.<br>No significant differences between the two groups immediately after awakening.<br>Negative correlation with the degree of depression in the study group<br>Increase after awakening and total output in 45th minute was significantly smaller in the study group than in the control group.<br>Negative correlation with both depressive mood status scales - BDI and HDRS
	Tene et al., 2018 [144]	NWS	N.d.<br>-80°C	Radioimmunoassay	Elevated bedtime levels significantly correlated with a greater neurological deficit, poorer white matter lesion score, more slender parietal cortex, smaller volume of white matter, poorer global microstructural integrity of white matter and hippocampus<br>Bedtime levels not correlated with the volume of ischemic lesions or total intracranial volume.<br>Morning levels not correlated with brain alterations in neuroimaging.<br>Elevated rates in all patients at the baseline than during follow-up visits (6, 12, 24 months later).<br>The high cortisol subgroup rates remained higher than rates from the low cortisol subgroup at 6, 12, and 24 months after stroke compared to admission levels.<br>The high cortisol subgroup admission rates exhibited significantly smaller total white and gray matter as well as hippocampal volumes, greater lesion volume of white matter, and poorer global microstructural integrity of

					white matter than the low cortisol subgroup<br>Higher bedtime levels significantly correlated with poorer executive function and attention scores on admission, 6, 12, and 24 months after stroke, as well as with worse global computerized cognitive scores 12 and 24 months after stroke.<br>Significant differences in executive function and memory scores - in the low cortisol subgroup the improvement maintained 24 months after stroke, while in the high cortisol subgroup cognitive decline was observed.<br>higher bedtime levels correlated with lower visuospatial function 12 months after stroke and with poorer global cognitive score, motor skills and memory 24 months after stroke.<br>Morning levels not correlated with cognitive rates.
	Mirzaee et al., 2019 [143]	NWS	N.d.<br>-80°C	The human saliva cortisol EIA kit	Significantly higher in the NWS of the study group than the control group at baseline.<br>Study group: decrease after stress, but not significant.
	Wang et al., 2021 [146]	NWS	N.d.<br>-20°C	Electrochemiluminescence immunoassay	Significantly higher in the NWS of CIS-MCI than in the CIS group<br><br>Patient age, hyperlipidemia history, TC, LDL-C, and MMSE were significantly different within various quartiles according to univariate analysis<br><br>Age, hyperlipidemia, history of hyperlipidemia, TC, LDL-C significantly increased with increasing cortisol levels<br>MMSE scores presented no significant differences according to pairwise comparison<br>Univariate and multivariate logistic regression analyses revealed that the MCI risk of patients in the first quartile was 0.35 and 0.41

					times of that in the fourth quartile, respectively.
	Zhanina et al., 2022 [147]	N.d.	2000× g for 15 min	enzymatically amplified chemiluminescence	Significantly higher in study group compared to control group 30, 180 and 365 (maximum level) days after stroke<br>Significant increase 365 days after stroke compared to acute stroke period
	Atam et al., 2025 [151]	N.d.	N.d.	ELISA	Higher in the study group compared to controls, however not significant difference
CRP	Malik et al., 2019 [58]	GCF	N.d.<br>-80°C	ELISA	Significant correlation with GCF IL-6<br>Significant correlation with age, dental plaque, functional dependency levels<br>Positive correlation between dental plaque scores and CRP
	Haba et al., 2011 [119]	GCF	N.d.<br>-20°C	ELISA	Significantly higher in patients with periodontal disease group<br>No significant correlations
Dityrosine	Maciejczyk et al., 2020 [61]	NWS/SWS	3000xg, 20 min, + 4 °C<br>-80°C	Fluorometrically	Significantly higher in the NWS of the HS subgroup than the NS subgroup and the control group<br>No significant differences in the SWS between groups
basic FGF	Forszt et al., 2025 [115]	NWS	3000xg, 20 min, + 4 °C<br>-80°C	immunofluorometric assay	Significantly higher in NWS of the study group than the control group<br>Negative correlation with ACE-R<br>100% sensitivity and specificity
Formaldehyde	Tong et al. 2017 [180]	NWS	8000×g, 10 min, + 4 °C<br>–70◦C	Fluo-HPLC	No significant correlations between formaldehyde levels in the NWS and ADL, CDR and MMSE scores
GSH	Al-Rawi et al., 2008 [95]	NWS	3000xg, 5 min, + 4 °C<br>-20°C	Burtis & Ashwood method	No significant differences between the groups

G-CSF	Forszt et al., 2025 [115]	NWS	3000xg, 20 min, + 4 °C -80°C	immunofluorometric assay	Significantly higher in NWS of the study group than the control group<br>Positive correlation with FIM<br>Positive correlation with SBS<br>Positive correlation with BI
GM-CSF	Forszt et al., 2025 [115]	NWS	3000xg, 20 min, + 4 °C -80°C	immunofluorometric assay	Below detection level
HGF	Forszt et al., 2025 [115]	NWS	3000xg, 20 min, + 4 °C -80°C	immunofluorometric assay	Significantly higher in NWS of the study group than the control group<br>100% sensitivity and specificity
H_2O_2	Maciejczyk et al., 2022 [69]	NWS/SWS	3000xg, 20 min, + 4 °C -80°C	Stoichiometrically	Significantly higher in the SWS of the hemorrhagic subgroup than the control group<br>Significantly higher in the NWS/SWS of the ischemic subgroup than the control group
IFN-α2	Forszt et al., 2025 [114]	NWS	3000xg, 20 min, + 4 °C -80°C	immunofluorometric assay	Below detection level
IFN-γ	Forszt et al., 2025 [114]	NWS	3000xg, 20 min, + 4 °C -80°C	immunofluorometric assay	Significantly higher in the NWS of the study group than the control group<br>Negative correlation with ACE-R<br>100% sensitivity and specificity
IL-1α	Forszt et al., 2025 [114]	NWS	3000xg, 20 min, + 4 °C -80°C	immunofluorometric assay	Significantly lower in NWS of the study group than the control group<br>Positive correlation with PI
IL-1β	Palm et al., 2014 [127]	SWS	N.d.<br>−70℃	ELISA	Significantly higher in the SWS of the control group than the study group
	Pawlukowska et al., 2022 [126]	NWS	N.d.<br>−70℃	ELISA	No significant differences between the groups
	Forszt et al., 2025 [114]	NWS	3000xg, 20 min, + 4 °C -80°C	immunofluorometric assay	Significantly higher in NWS of the study group than the control group<br>Negative correlation with ACE-R<br>Positive correlation with FIM<br>Positive correlation with SBS
IL-1ra	Forszt et al., 2025 [114]	NWS	3000xg, 20 min, + 4 °C -80°C	immunofluorometric assay	Significantly higher in the NWS of the study group than the control group<br>A significant correlation with ACE-R

IL-2	Forszt et al., 2025 [114]	NWS	3000xg, 20 min, + 4 °C<br>-80°C	immunofluorometric assay	Below detection level
IL-2Rα/CD25	Forszt et al., 2025 [114]	NWS	3000xg, 20 min, + 4 °C<br>-80°C	immunofluorometric assay	Significantly higher in the NWS of the study group than the control group
IL-3/MCGF	Forszt et al., 2025 [114]	NWS	3000xg, 20 min, + 4 °C<br>-80°C	immunofluorometric assay	Significantly lower in NWS of the study group than the control group<br>Positive correlation with FIM<br>100% sensitivity and specificity
IL-4	Forszt et al., 2025 [114]	NWS	3000xg, 20 min, + 4 °C<br>-80°C	immunofluorometric assay	Significantly lower in the NWS of the study group than the control group
IL-5	Forszt et al., 2025 [114]	NWS	3000xg, 20 min, + 4 °C<br>-80°C	immunofluorometric assay	Below detection level
IL-6	Malik et al., 2019 [58]	GCF	N.d.<br>-80°C	ELISA	No significant correlation with dental plaque, functional dependency levels<br>Positive correlation with swallowing problems<br>High levels correlated with right hemiparesis<br>Decreased levels correlated with multiple comorbidities
	Maciejczyk et al., 2021 [12]	NWS/SWS	3000xg, 20 min, + 4 °C<br>-80°C	ELISA	Significantly higher in the NWS/SWS of the study group than the control group
	Haba et al., 2011 [119]	GCF	N.d.<br>-20°C	ELISA	Significant correlation (weak) with serum CRP in patients with periodontal disease group and patients with periodontal disease and transient ischemic attack TIA group<br>Significant correlation with serum IL-6 in patients with periodontal disease group and patients with periodontal disease and transient ischemic attack TIA group
	Forszt et al., 2025 [114]	NWS	3000xg, 20 min, + 4 °C<br>-80°C	immunofluorometric assay	Significantly higher in the NWS of the study group than the control group<br>Positive correlation with FIM<br>Positive correlation with SBS<br>A significant correlation with ACE-R<br>Positive correlation with PI
IL-7	Forszt et al., 2025 [114]	NWS	3000xg, 20 min, + 4 °C<br>-80°C	immunofluorometric assay	Below detection level

IL-9	Forszt et al., 2025 [114]	NWS	3000xg, 20 min, + 4 °C -80°C	immunofluorometric assay	Significantly lower in the NWS of the study group than the control group
IL-10	Maciejczyk et al., 2021 [12]	NWS/SWS	3000xg, 20 min, + 4 °C -80°C	ELISA	Significantly lower in the NWS/SWS of the study group than the control group
	Forszt et al., 2025 [114]	NWS	3000xg, 20 min, + 4 °C -80°C	immunofluorometric assay	Below detection level
IL-12 p40	Forszt et al., 2025 [114]	NWS	3000xg, 20 min, + 4 °C -80°C	immunofluorometric assay	Significantly higher in the NWS of the study group than the control group<br>A significant correlation with FIM<br>100% sensitivity and specificity
IL-12 p70	Forszt et al., 2025 [114]	NWS	3000xg, 20 min, + 4 °C -80°C	immunofluorometric assay	Below detection level
IL-13	Forszt et al., 2025 [114]	NWS	3000xg, 20 min, + 4 °C -80°C	immunofluorometric assay	Below detection level
IL-15	Forszt et al., 2025 [114]	NWS	3000xg, 20 min, + 4 °C -80°C	immunofluorometric assay	Below detection level
IL-16	Forszt et al., 2025 [114]	NWS	3000xg, 20 min, + 4 °C -80°C	immunofluorometric assay	No significant differences between the groups
IL-17	Forszt et al., 2025 [114]	NWS	3000xg, 20 min, + 4 °C -80°C	immunofluorometric assay	No significant differences between the groups<br>A significant correlation with FIM
IL-18	Forszt et al., 2025 [114]	NWS	3000xg, 20 min, + 4 °C -80°C	immunofluorometric assay	No significant differences between the groups<br>A significant correlation with ACE-R
Iron	García-Cabo et al.2020 [99]	NWS	N.d.	ICP-MS	No significant differences between the groups
Kynurenine	Maciejczyk et al., 2020 [61]	NWS/SWS	3000xg, 20 min, + 4 °C -80°C	Fluorometrically	Significantly higher in the NWS of HS subgroup than the NS subgroup and the control group<br>No significant differences in the SWS between the groups
LIF	Forszt et al., 2025 [115]	NWS	3000xg, 20 min, + 4 °C -80°C	immunofluorometric assay	Significantly higher in NWS of the study group than the control group<br>Positive correlation with FIM<br>100% sensitivity and specificity
M-CSF	Forszt et al., 2025 [115]	NWS	3000xg, 20 min, + 4 °C -80°C	immunofluorometric assay	No significant differences between the groups<br>Negative correlation with ACE-R

MIF	Forszt et al., 2025 [115]	NWS	3000xg, 20 min, + 4 °C -80°C	immunofluorometric assay	Significantly higher in NWS of the study group than the control group
MDA	Al-Rawi et al., 2008 [98]	NWS	3000xg, 5 min, + 4 °C -20°C	Buege & Aust method, spectrophotometric	Significantly higher in the NWS of the study group than the control group<br>Positive correlation with salivary UA
	Al-Rawi, 2010 [96]	NWS	3000xg, 5 min, + 4 °C -20°C	Buege & Aust method, spectrophotometric	Significantly higher in the NWS of the study group than the control group<br>Positive correlation with LDL-C, TAG in serum and saliva
MMP-8	Palm et al., 2014 [127]	SWS	N.d.<br>−70℃	Time-resolved immunofluorometric assay	Significantly lower in the SWS of the study group than the control group
	Pawlukowska et al., 2022 [123]	NWS	N.d.<br>−70℃	Immunofluorescence	No significant differences between the groups
Melatonin	Sun et al., 2026 [176]	NWS	N.d.	ELISA	Phase 1:<br>Significantly lower in the NWS of the study group than the control group in all time points (0:00, 3:00, 6:00, 18:00, and 21:00)<br>Significantly lower AUC in the study group than the control group<br>Lower amplitude and peak in the AIS group<br>The peak time was delayed - in the AIS group was 03:00, in the control group was 00:00<br><br>Phase 2:<br>Significantly lower salivary melatonin levels at 19:00–23:00 in AIS patients compared to controls<br>Significantly lower melatonin area under the curve (AUC) values across all time intervals in AIS patients<br>Significantly delayed dim light melatonin onset (DLMO) in the AIS group (94 minutes)<br>Significantly reduced post-DLMO surge in AIS patients<br>Significantly lower AUC30 in AIS patients compared to controls<br>Significantly lower levels at 22:00 in AIS patients with poor prognosis than in those with good prognosis

					Delayed DLMO was an independent risk<br>factor for poor prognosis in AIS patients
Microbiological analysis	Cieplik et al., 2020 [157]	Samples from the dorsal surface of the tongue and subgingival plaque/alveolar ridges	N.d.<br>−80◦C	Culture technique + MALDI-TOF MS	Detection:<br>Rothia mucilaginosa 76.8%<br>Viridans streptococci 75.8%<br>Prevotella melaninogenica 74.7% Streptococcus oralis 61.6%<br>Neisseria spp. 59.6%<br>No significant differences between the groups at the baseline<br>Decrease only in Rothia spp by >20% in C+S groups over time<br>Decrease in Rothia, Streptococcus, Prevotella, Neisseria, Haemophilus, and Leptotrichia spp by >20% each in P group over time<br>Increase in Klebsiella,Staphylococcus and Candida spp. by >20% each in P group over time
				16S rRNA amplicon sequencing.	No significant differences in species richness between the groups at the baseline<br>No significant differences in microbial compositions between C group and P group at the baseline<br>No significant differences in microbial compositions between C group and stroke group<br>Enrichment of Neisseria, Porphyromonas and Prevotella in P group compared to C group at the baseline<br>Loss of Streptococcus spp. in P group compared to C group at the baseline<br>Significantly higher oral clone AG008 Streptococcus sp. in P group after 120h than in P group at the baseline<br>Significantly lower Actinomyces, Leptotrichia, Fusobacterium, Prevotella, Haemophilus, Neisseria, and Streptococcus spp. in P group after 120h than in P group at the baseline

	Inenaga et al., 2018 [158]	NWS	N.d.	Culture technique+PCR	Significantly higher Cnm-positive/SM-positive ratio in the NWS of CES group, CES/NVAF group, non-CES infarct group, ICH group and RIA group than the control group<br>No significant differences in isolation rate of SM between the groups
	Manzoor et al., 2024 [159]	SWS	8000rpm, 1 min, -70°C	Spectrophotometrically/Fluorometrically	No significant difference in microbial community composition (beta diversity) between study group and controls<br>Oral microbiome was dominated by the phyla Bacillota, Actinomycetota, Bacteroidota, Pseudomonadota and Fusobacteriota, accounting for >98% of the salivary microbiota<br>The most abundant bacterial genera were Prevotella, Streptococcus, Neisseria, Veillonella and Schaalia<br>Archaea were detected in saliva, with dominant genera Halobaculum, Halorubrum, Methanosarcina, Natrinema, and Halobacterium<br>Fungi were detected in saliva, with dominant genera Fusarium, Candida, Aspergillus, Pyricularia, and Saccharomyces<br>Viruses were detected in saliva, with dominant genera Moineauvirus, Roseolovirus, Dybvigvirus, Brussowvirus, and Cepunavirus<br>Significantly higher abundance of bacterial species Brenneria goodwinii, Variovorax boronicumulans, Pseudomonas sp. AN-B15, Actinoalloteichus sp. GBA129-24 and Thiomonas arsenitoxydans in the study group compared to controls<br>Significantly lower abundance of bacterial species Metamycoplasma alkalescens, Streptomyces sp. LBUM 1475, Pseudonocardia dioxanivorans, Arthrobacter dokdonella and Francisella frigiditurris in the study group compared to controls

					Significantly lower abundance of viral species Brussowvirus ALQ132 and Moineauvirus Abc2 in the study group compared to controls<br>Five metagenomic pathways were significantly different between the study group and controls<br>Significantly increased abundance of inosine 5′-phosphate biosynthesis II and III and stachyose degradation pathways in the study group compared to controls<br>Microbial interaction networks showed significantly higher correlations and increased species clustering in patients compared to controls
	Roongpiboonsopit et al., 2025 [162]	NWS	5,000×g, 5 min<br>−20℃	PCR	Significantly higher relative abundance of phylum Bacillota in stroke individuals compared to controls<br>Significantly lower relative abundance of Bacteroidota, Actinomycetota and Pseudomonadota in stroke individuals<br>Slightly higher prevalence of Fusobacteria in stroke individuals<br>Significantly different overall salivary microbiota composition at the genus level between control and stroke groups<br>Significantly higher abundance of Streptococcus in the saliva of stroke individuals<br>Lower abundance of Prevotella in the saliva of stroke individuals<br>Significantly increased relative abundance of Streptococcus infantis and decreased relative abundance of Prevotella melaninogenica in stroke individuals<br>Significantly higher number of OTUs and significantly increased Chao1 richness in stroke patients, indicating greater microbial diversity<br>Significant OTU-based microbial biomarkers distinguishing control and stroke groups, with Prevotella

					melaninogenica, Neisseria subflava, Veillonella parvula and Haemophilus parainfluenzae significantly enriched in controls, and Granulicatella spp., Porphyromonas endodontalis, Rothia dentocariosa, Streptococcus anginosus, Prevotella nigrescens and Atopobium rimae significantly enriched in stroke individuals<br>Significantly altered microbial functional profiles in stroke patients, with enrichment of membrane transport, xenobiotics biodegradation and metabolism, and cardiovascular disease–related pathways, while controls showed significantly higher replication and repair, energy metabolism, and cellular processes
	Tonomura et al., 2025 [161]	NWS	N.d.<br>−80℃	spectrophotometrically	No significant difference in overall salivary microbiome diversity between acute stroke patients and non-stroke participants<br>Significantly lower gut microbiome diversity in patients with acute stroke compared to non-stroke participants<br>Higher abundance of Treponema denticola, Streptococcus cristatus, Prevotella loescheii and Streptococcus anginosus in the salivary microbiome of acute stroke patients<br>Higher abundance of Eubacterium sulci, Streptococcus infantis, Haemophilus parainfluenzae, Prevotella nanceiensis and Lancefieldella parvula in the salivary microbiome of non-stroke participants<br>Significantly higher proportion of Streptococcus anginosus in both the salivary and gut microbiomes of patients with acute stroke compared to non-stroke participants<br>Presence of Streptococcus anginosus in the gut microbiome was independently and significantly associated

					with stroke, prognostic value for mortality and cardiovascular events, based on multivariable analysis
	Manzoor et al., 2025 [160]	SWS	8000rpm, 1 min, -70°C	Spectrophotometrically/Fluorometrically	Oral microbiota was dominated by the phyla Bacillota, Actinomycetota, Bacteroidota, Pseudomonadota and Fusobacteriota<br>The most abundant genera were Prevotella, Streptococcus, Neisseria, Veillonella, Schaalia and Haemophilus<br>No significant difference in alpha or beta diversity of the oral microbiota between CIS patients with high-risk PFO and those without PFO<br>Differences in relative abundance of major phyla between cryptogenic ischemic stroke patients with and without high-risk PFO were observed, but not statistically significant<br>Ascomycota was significantly positively associated with cryptogenic ischemic stroke patients with high-risk PFO after adjustment for age, hypertension and heavy alcohol use<br>At the class level, Saccharomycetes was significantly more abundant in cryptogenic ischemic stroke patients with high-risk PFO<br>No genera or species showed statistically significant differences between cryptogenic ischemic stroke patients with and without high-risk PFO in the adjusted model<br>No significant difference in alpha diversity between cryptogenic ischemic stroke patients and controls with high-risk PFO<br>Significantly different beta diversity of the oral microbiome between cryptogenic ischemic stroke patients and controls with high-risk PFO<br>Significantly higher abundance of phyla Bacteroidota and Synergistota in cryptogenic ischemic stroke patients

					compared to controls with high-risk PFO<br>Significantly higher abundance of the order Desulfobacterales in cryptogenic ischemic stroke patients<br>Significantly lower abundance of the genus Lactococcus and species Lactococcus raffinolactis and Lactococcus cremoris in cryptogenic ischemic stroke patients compared to controls
MPO	Palm et al, 2014 [127]	SWS	N.d.<br>−70℃	ELISA	Significantly lower in the SWS of the study group than the control group
NGF-β	Forszt et al., 2025 [115]	NWS	3000xg, 20 min, + 4 °C -80°C	immunofluorometric assay	Below detection level
NSE	Al-Rawi et al., 2009 [128]	NWS	3000xg, 5 min, + 4 °C -20°C	Spectrophotometrically	Significantly higher in the NWS of the study group than the controls<br>No significant differences between the study group and the risk groups
NO	Maciejczyk et al., 2020 [61]	NWS/SWS	3000xg, 20 min, + 4 °C -80°C	Colorimetrically	Significantly lower in the NWS of the HS subgroup than the NS subgroup and the control group<br>No significant differences in the SWS between the groups
Nitrotyrosine concentration	Maciejczyk et al., 2020 [61]	NWS/SWS	3000xg, 20 min, + 4 °C -80°C	Colorimetrically	Significantly higher in the NWS of the HS subgroup than the NS subgroup and the control group<br>No significant differences in the SWS between the groups
N-formylkynurenine	Maciejczyk et al., 2020 [61]	NWS/SWS	3000xg, 20 min, + 4 °C -80°C	Fluorometrically	Significantly higher in the NWS of the HS subgroup than the NS subgroup and the control group<br>No significant differences in the SWS between the groups
Osteoprotegerin	Pawlukowska et al., 2022 [126]	NWS	N.d.<br>−70℃	ELISA	No significant differences between the groups
OSI	Gerreth et al., 2020 [13]	NWS/SWS	3000xg, 20 min, + 4 °C -80°C	TOS)/ TACx100	Significantly higher in the SWS of the study group than the control group
Px	Gerreth et al., 2020 [13]	NWS/SWS	3000xg, 20 min, + 4 °C -80°C	Colorimetrically	Significantly higher in the NWS/SWS of the study group than the control group

Peroxynitrite	Maciejczyk et al., 2020 [61]	NWS/SWS	3000xg, 20 min, + 4 °C<br>-80°C	Colorimetrically	Significantly higher in the NWS of both subgroups than the control group<br>Significantly higher in the SWS of the HS subgroup than the NS subgroup and the control group
PDGF-BB	Forszt et al., 2025 [115]	NWS	3000xg, 20 min, + 4 °C<br>-80°C	immunofluorometric assay	Significantly lower in NWS of the study group than the control group
PC	Maciejczyk et al., 2020 [61]	NWS/SWS	3000xg, 20 min, + 4 °C<br>-80°C	Colorimetrically	Significantly higher in the NWS of the HS subgroup than the control group<br>No significant differences in the SWS between the groups
RANKL	Pawlukowska et al., 2022 [126]	NWS	N.d.<br>−70℃	ELISA	No significant differences between the groups
Reduced glutathione	Gerreth et al., 2020 [13]	NWS/SWS	3000xg, 20 min, + 4 °C<br>-80°C	Colorimetrically	Significantly lower in the SWS of the study group than the control group
SCGF-β	Forszt et al., 2025 [115]	NWS	3000xg, 20 min, + 4 °C<br>-80°C	immunofluorometric assay	Below detection level
SCF	Forszt et al., 2025 [115]	NWS	3000xg, 20 min, + 4 °C<br>-80°C	immunofluorometric assay	Below detection level
SOD	Al-Rawi et al., 2008 [95]	NWS	3000xg, 5 min, + 4 °C<br>-20°C	Photochemical Nitroblue Tatrazolium method	Significantly higher in the NWS of the study group than the control group<br>Positive correlation with salivary UA
	Gerreth et al., 2020 [13]	NWS/SWS	3000xg, 20 min, + 4 °C<br>-80°C	Colorimetrically	Significantly higher in the NWS of the study group than the control group
SP	Muhle et al., 2017 [172]	NWS	4110 g,5 min<br>-20°C	ELISA-type immunoassay	Post- over pre-stimulation increase was correlated with successful PES<br>Small decrease was correlated with non-successfully PES<br>During the first cycle greater increase after the second stimulation (+ 28%) than after the third stimulation (+ 11%)<br>No increase after the first stimulation<br>No increase of mean SP level in patients with treatment failure during three sessions

	Niimi et al., 2018 [173]	NWS	N.d.<br>-80°C	SP Parameter Assay Kit	Significantly lower frequency of spontaneous swallowings in the low SP group than high SP group<br>Significantly more frequent the incidence of pneumonia in the low SP group than high SP group
	Alvarez-Larruy et al., 2023 [174]	NWS	2600 rpm,2 min<br>-80°C	SP Parameter Assay Kit	No significant differences between the groups in the morning and in the afternoon
TIMP-1	Palm et al., 2014 [127]	SWS	N.d.<br>−70℃	ELISA	No significant differences between the groups
TAC	Gerreth et al., 2020 [13]	NWS/SWS	3000xg, 20 min, + 4 °C<br>-80°C	Colorimetrically	No significant differences between the groups
TOS	Gerreth et al., 2020 [13]	NWS/SWS	3000xg, 20 min, + 4 °C<br>-80°C	Bichromatically	Significantly higher in the NWS/SWS of the study group than the control group
TPC	Gerreth et al., 2020 [13]	NWS/SWS	3000xg, 20 min, + 4 °C<br>-80°C	Colorimetrically	No significant difference in the NWS<br>Significantly lower in the SWS in the study group than the control group
	Maciejczyk et al., 2020 [61]	NWS/SWS	3000xg, 20 min, + 4 °C<br>-80°C	Colorimetrically	Significantly decreased in the hyposalivation subgroup of stroke patients compared with normal salivation subgroup and control group in NWS, SWS
	Maciejczyk et al., 2021 [12]	NWS/SWS	3000xg, 20 min, + 4 °C<br>-80°C	Colorimetrically	No significant difference in the NWS<br>Significantly lower in the SWS in the study group than the control group
	Maciejczyk et al., 2022 [69]	NWS/SWS	3000xg, 20 min, + 4 °C<br>-80°C	Colorimetrically	Significantly lower in the NWS/SWS of both study subgroups than the control group
Total thiols	Maciejczyk et al., 2020 [61]	NWS/SWS	3000xg, 20 min, + 4 °C<br>-80°C	Ellman's reagent	Significantly lower in the NWS/SWS of both subgroups than the control group
Tryptophan	Maciejczyk et al., 2020 [61]	NWS/SWS	3000xg, 20 min, + 4 °C<br>-80°C	Fluorometrically	Significantly lower in the NWS/SWS of the HS subgroup than the control group<br>Significantly lower in the NWS of the HS subgroup than the NS subgroup

TNF-α	Maciejczyk et al., 2021 [12]	NWS/SWS	3000xg, 20 min, + 4 °C -80°C	ELISA	Significantly higher in the NWS/SWS of the study group than the control group
	Forszt et al., 2025 [114]	NWS	3000xg, 20 min, + 4 °C -80°C	immunofluorometric assay	Significantly higher in NWS of the study group than the control group<br>Negative correlation with ACE-R<br>Positive correlation with PI<br>100% sensitivity and specificity
TNF-β	Forszt et al., 2025 [114]	NWS	3000xg, 20 min, + 4 °C -80°C	immunofluorometric assay	Significantly higher in NWS of the study group than the control group<br>Negative correlation with ACE-R<br>A significant correlation with FIM<br>Positive correlation with PI<br>100% sensitivity and specificity
TRAIL	Forszt et al., 2025 [114]	NWS	3000xg, 20 min, + 4 °C -80°C	immunofluorometric assay	Significantly higher in the NWS of the study group than the control group
UA	Al-Rawi et al., 2008 [95]	NWS	3000xg, 5 min, + 4 °C -20°C	BioMerieux, France kit	Significantly higher in the NWS of the study group than the control group<br>Positive correlation with salivary MDA,SOD
	Gerreth et al., 2020 [13]	NWS/SWS	3000xg, 20 min, + 4 °C -80°C	Colorimetrically	Significantly higher in the NWS/SWS of the study group than the control group
	Maciejczyk et al., 2022 [69]	NWS/SWS	3000xg, 20 min, + 4 °C -80°C	Colorimetrically	Significantly higher in the NWS of the hemorrhagic subgroup than the control group<br>Significantly higher in the NWS of the ischemic subgroup than the control group
VEGF	Forszt et al., 2025 [115]	NWS	3000xg, 20 min, + 4 °C -80°C	immunofluorometric assay	Significantly higher in NWS of the study group than the control group<br>Negative correlation with ACE-R
XO activity	Maciejczyk et al., 2022 [69]	NWS/SWS	3000xg, 20 min, + 4 °C -80°C	Colorimetrically<br>Fluorometrically	Significantly higher XO activity in the NWS of the hemorrhagic subgroup than the control group<br>Significantly higher specific activity in the NWS/SWS of the hemorrhagic subgroup than the control group<br>Significantly higher XO activity, XO output in the NWS

					of the ischemic subgroup than the control group<br>Significantly higher specific activity in the NWS/SWS of the ischemic subgroup than the control group

ACE-R – Addenbrooke's Cognitive Examination Revised; ADL – Activities of Daily Living; AGE – Advanced Glycation End products; AIS – Acute Ischemic Stroke; AOPP – Advanced Oxidation Protein Products; BDI – Beck Depression Inventory II; BDNF – Brain-Derived Neurotrophic Factor; BI – Barthel Index; BP – Blood Pressure; C – Stroke Mimics Patients; CAT – Catalase; CCL2 / MCP-1 – Chemokine (C-C motif) Ligand 2 / Monocyte Chemoattractant Protein-1; CCL3 / MIP-1α – Chemokine (C-C motif) Ligand 3 / Macrophage Inflammatory Protein-1 alpha; CCL4 / MIP-1β – Chemokine (C-C motif) Ligand 4 / Macrophage Inflammatory Protein-1 beta; CCL5 / RANTES – Chemokine (C-C motif) Ligand 5 / Regulated on Activation, Normal T-cell Expressed and Secreted; CCL7 / MCP-3 – Chemokine (C-C motif) Ligand 7 / Monocyte Chemoattractant Protein-3; CCL11 / eotaxin – Chemokine (C-C motif) Ligand 11 / Eotaxin; CCL27 / CTACK – Chemokine (C-C motif) Ligand 27 / Cutaneous T-cell-Attracting Chemokine; CDR – Clinical Dementia Rating; CES – Cardioembolic Stroke; CIS – Cerebral Ischemic Stroke; CIS-MCI – Cerebral Ischemic Stroke with Mild Cognitive Impairment; CRP – C-Reactive Protein; CXCL1 / GRO-α – Chemokine (C-X-C motif) Ligand 1 / Growth-Regulated Alpha Protein; CXCL8 / IL-8 – Chemokine (C-X-C motif) Ligand 8 / Interleukin-8; CXCL9 / MIG – Chemokine (C-X-C motif) Ligand 9 / Monokine Induced by Gamma Interferon; CXCL10 / IP-10 – Chemokine (C-X-C motif) Ligand 10 / Interferon Gamma-Induced Protein 10; CXCL12 / SDF-1α – Chemokine (C-X-C motif) Ligand 12 / Stromal Cell-Derived Factor-1 alpha; DBP – Diastolic Blood Pressure; DLMO – Dim Light Melatonin Onset; EIA – Enzyme Immunoassay; FGF – Fibroblast Growth Factor (basic); FIM – Functional Independence Measure; Fluo-HPLC – High-Performance Liquid Chromatography with Fluorescence Detection; GCF – Gingival Crevicular Fluid; G-CSF – Granulocyte Colony-Stimulating Factor; GM-CSF – Granulocyte-Macrophage Colony-Stimulating Factor; GSH – Glutathione; H2O2 – Hydrogen Peroxide; HDRS – Hamilton Depression Rating Scale; HGF – Hepatocyte Growth Factor; HR – Heart Rate; HS – Hyposalivation; ICP-MS – Inductively Coupled Plasma Mass Spectrometry; ICH – Intracerebral Hemorrhage; IFN-α2 – Interferon Alpha-2; IFN-γ – Interferon Gamma; IL-1α – Interleukin-1 alpha; IL-1β – Interleukin-1 beta; IL-1ra – Interleukin-1 Receptor Antagonist; IL-2 – Interleukin-2; IL-2Rα / CD25 – Interleukin-2 Receptor Alpha / Cluster of Differentiation 25; IL-3 / MCGF – Interleukin-3 / Mast Cell Growth Factor; IL-4 – Interleukin-4; IL-5 – Interleukin-5; IL-6 – Interleukin-6; IL-7 – Interleukin-7; IL-9 – Interleukin-9; IL-10 – Interleukin-10; IL-12 p40 – Interleukin-12 p40 Subunit; IL-12 p70 – Interleukin-12 p70 Heterodimer; IL-13 – Interleukin-13; IL-15 – Interleukin-15; IL-16 – Interleukin-16; IL-17 – Interleukin-17; IL-18 – Interleukin-18; LDL-C – Low-Density Lipoprotein Cholesterol; LH – Left Hemisphere; LIF – Leukemia Inhibitory Factor; LOOH – Lipid Hydroperoxides; MALDI-TOF MS – Matrix-Assisted Laser Desorption Ionization–Time of Flight Mass Spectrometry; MCI – Mild Cognitive Impairment; M-CSF – Macrophage Colony-Stimulating Factor; MDA – Malondialdehyde; MEPs – Motor-Evoked Potentials; MIF – Macrophage Migration Inhibitory Factor; MMSE – Mini-Mental State Examination; MMP-8 – Matrix Metalloproteinase-8; MPO – Myeloperoxidase; NBT – Nitro Blue Tetrazolium; N.d. - No data;NGF-β – Nerve Growth Factor beta; NO – Nitric Oxide; NS – Normal Salivary Secretion; NSE – Neuron-Specific Enolase; NVAF – Nonvalvular Atrial Fibrillation; NWS – Non-Stimulated Whole Saliva; OSI – Oxidative Stress Index; OTUs – Operational Taxonomic Units; P – Stroke Patients with Pneumonia; PB – Practical Baseline; PC – Protein Carbonyls; PDGF-BB – Platelet-Derived Growth Factor, Isoform BB; PES – Pharyngeal Electrical Stimulation; PFO – Patent Foramen Ovale; PI – Plaque Index; Px – Peroxidase; RANKL – Receptor Activator of Nuclear Factor κB Ligand; RH – Right Hemisphere; RIA – Ruptured Intracranial Aneurysm; SBS – Sitting Balance Scale; SBP – Systolic Blood Pressure; SCF – Stem Cell Factor; SCGF-β – Stem Cell Growth Factor beta; SM – Streptococcus mutans; SOD – Superoxide Dismutase; SP - Substance P; SWS – Stimulated Whole Saliva; TAG – Triacylglycerols; TAC – Total Antioxidant Capacity; TC – Total Cholesterol; TIMP-1 – Tissue Inhibitor of Metalloproteinases-1; TNF-α – Tumor Necrosis Factor alpha; TNF-β – Tumor Necrosis Factor beta; TOS –

Total Oxidant Status; TPC – Total Protein Content; TRAIL – Tumor Necrosis Factor-Related Apoptosis-Inducing Ligand; UA – Uric Acid; VEGF – Vascular Endothelial Growth Factor; WAB-AQ – Western Aphasia Battery Aphasia Quotient; XO – Xanthine Oxidase.
